# Discovery of Natural α-Glucosidase Inhibitors from *Hericium erinaceus* Through Integrated Isolation, Structural Characterization, In Vitro Evaluation, and Molecular Dynamics Simulations

**DOI:** 10.3390/molecules31101605

**Published:** 2026-05-11

**Authors:** Xianxian Miao, Xiangming Kong, Xiaodong Shang, Yan Yang, Wei Han, Jingsong Zhang, Na Feng

**Affiliations:** 1 Institute of Edible Fungi, Shanghai Academy of Agricultural Sciences, Shanghai 201403, China; mrx@saas.sh.cn (X.M.); shangxiaodong@saas.sh.cn (X.S.); yangyan@saas.sh.cn (Y.Y.); 2 Shanghai Key Laboratory of New Drug Design, Engineering Research Center of Pharmaceutical Process Chemistry, Ministry of Education, School of Pharmacy, East China University of Science and Technology, Shanghai 200237, China; 20240505@saas.sh.cn

**Keywords:** *Hericium erinaceus*, isoindolin-1-one compounds, *α*-glucosidase activity assay, molecular dynamics simulation, surface plasmon resonance assay

## Abstract

Recognized for its dual nutritional and therapeutic value, the fungus *Hericium erinaceus* is increasingly acknowledged as a rich resource of naturally derived *α*-glucosidase inhibitors. In this study, eight compounds were isolated from *H. erinaceus*, including three novel compounds designated as erinacerins X−Z (**1**, **2**, and **6**). Their absolute configurations were definitively elucidated using a combination of NMR, HR-MS, and ECD calculations. Furthermore, an integrated screening strategy combining molecular docking, molecular dynamics (MD) simulations, and surface plasmon resonance (SPR) analysis identified two isoindolin-1-ones (**2** and **3**) as potent naturally derived *α*-glucosidase inhibitors. Notably, in vitro testing established compounds **2** and **3** as robust *α*-glucosidase inhibitors, affording IC_50_ values of 17.80 ± 1.03 μM and 19.50 ± 1.33 μM, respectively. MD simulations revealed that electrostatic interactions and van der Waals forces are the primary drivers of this intermolecular association. These findings were further corroborated by SPR analysis, which quantified their high-affinity binding kinetics to the enzyme. Overall, this combined approach establishes a solid foundation for the discovery and development of natural *α*-glucosidase inhibitors from *H. erinaceus*.

## 1. Introduction

Widely recognized for its nutritional and therapeutic value, *Hericium erinaceus* (commonly referred to as the monkey head mushroom) is a well-known edible–medicinal fungus classified within the family *Hericiaceae* [1]. Phytochemical investigations have revealed that *H. erinaceus* mycelia yield a diverse array of secondary metabolites, including polysaccharides, erinacines, hericenones, isobenzofuranones, and ergosterols [2]. These constituents exhibit a broad spectrum of pharmacological properties, notably *α*-glucosidase inhibitory, antibacterial, neurotrophic, anti-inflammatory, and antitumor activities [3,4]. To date, approximately 150 small-molecule metabolites have been isolated and structurally characterized from this fungus.

Diabetes mellitus, a highly prevalent chronic endocrine disorder, is projected to affect 783.2 million people worldwide by 2045, imposing a severe economic burden on global healthcare systems [5]. Representing the vast majority (>90%) of clinical diagnoses, type 2 diabetes mellitus (T2DM) is mechanistically driven by a combination of systemic insulin resistance and the gradual decline in pancreatic insulin secretion [6]. Consequently, targeting carbohydrate-hydrolyzing enzymes, particularly *α*-glucosidase, has emerged as a leading therapeutic strategy for T2DM management [7,8]. Localized at the brush border membrane of intestinal epithelial cells, *α*-glucosidase catalyzes the rate-limiting hydrolysis of complex carbohydrates (e.g., polysaccharides and oligosaccharides) into absorbable monosaccharides [9]. Inhibiting this critical enzyme effectively delays intestinal carbohydrate absorption, thereby significantly reducing postprandial hyperglycemia [10]. However, conventional synthetic *α*-glucosidase inhibitors like acarbose are frequently associated with adverse gastrointestinal effects [11]. This limitation has driven extensive research into safer, naturally derived alternatives.

Previous investigations into the anti-diabetic potential of *H. erinaceus* have predominantly focused on extracts obtained using a highly polar solvent [4]. For example, the high-molecular-weight *H. erinaceus* polysaccharides (HEPs) enriched in aqueous extracts exhibit significant hypoglycemic potential, functioning primarily by modulating glucose metabolism-related enzymes or activating insulin signaling cascades, such as the IRS/PI3K/AKT pathway [12,13,14]. Furthermore, ethanolic and methanolic extracts, rich in phenolic compounds and moderately polar constituents, have also been widely validated for their hypoglycemic effects [15,16,17]. However, as research has progressed, analytical focus has increasingly shifted toward less polar extracts, notably the ethyl acetate (EtOAc) fraction, to discover direct enzyme inhibitors. Studies demonstrate that a diverse array of small-molecule secondary metabolites isolated from this fraction—including alkaloids (erinacerins M–P) [18], meroterpenoids (hericene B, hericenones C, E–G) [19], isoindolinone-containing meroterpene dimers (caputmedusins A–C) [20], and other structural variants (e.g., erinacenol D and hericene derivatives) [10]—exhibit varying degrees of direct inhibitory activity against *α*-glucosidase. Among these, a class of isoindolin-1-one derivatives known as erinacerins C–L has garnered considerable attention, positioning them as highly promising candidates for anti-diabetic therapeutics and functional foods [21,22].

**Figure 1:**
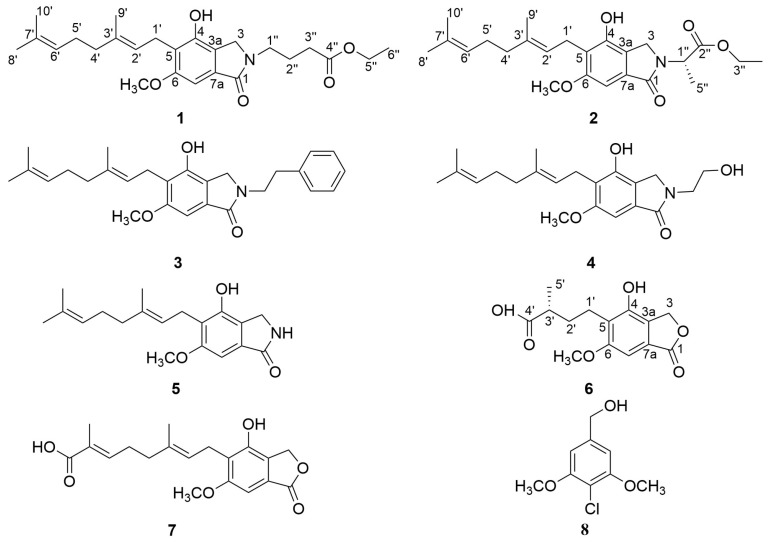
Structures of compounds **1**–**8**.

To identify novel natural *α*-glucosidase inhibitors, a phytochemical investigation of EtOAc extract from *H. erinaceus* mycelia was conducted, yielding three previously undescribed compounds, designated as erinacerins X–Z (**1**, **2**, and **6**), alongside five known analogues (Figure 1). The structures of these unreported isolates were established by nuclear magnetic resonance (NMR) analyses, high-resolution mass spectrometry (HR-MS), and theoretical electronic circular dichroism (ECD) simulations. Based on these structural findings, we established an integrated screening platform that incorporates in vitro enzymatic assays, molecular docking, molecular dynamics (MD) simulations, and surface plasmon resonance (SPR). Utilizing this systematic approach, two isoindolin-1-one derivatives (**2** and **3**) were successfully validated as potent *α*-glucosidase inhibitors. Ultimately, this combinatorial strategy not only accelerates the targeted discovery of *α*-glucosidase inhibitors from *H. erinaceus* but also provides a robust scientific foundation for elucidating their molecular mechanisms.

## 2. Results and Discussion

### 2.1. Isolation and Identification of Compounds from H. erinaceus

To discover natural *α*-glucosidase inhibitors from *H. erinaceus*, a static liquid fermentation strategy was employed to cultivate the mycelia of strain No. 7080. The resulting EtOAc extract underwent systematic fractionation via MPLC. Subsequent targeted purification, utilizing a combination of reversed-phase preparative HPLC, gel permeation chromatography, and recrystallization, successfully yielded eight individual metabolites (**1**–**8**). The comprehensive structural elucidation of these isolates is presented below.

Erinacerin X (**1**), with the molecular formula C_25_H_35_NO_5_ and nine double-bond equivalents (DBEs), was elucidated from the HRESIMS molecular ion peak at *m*/*z* 452.2404 [M + Na]^+^ (calcd for C_25_H_35_NO_5_Na, 452.2407). The FTIR spectrum demonstrated pronounced absorption bands at 3415 and 1668 cm^−1^, thereby substantiating the existence of -OH and C=O functional groups. The ^1^H NMR spectral data (Table 1) for **1** displayed an aromatic proton at *δ*_H_ 6.97 (1H, s), two olefinic protons at *δ*_H_ 5.25 (1H, t, *J* = 12.0 Hz) and *δ*_H_ 5.03 (1H, t, *J* = 6.0 Hz), a methoxy group at *δ*_H_ 3.86 (3H, s), eight methylene groups at *δ*_H_ 4.28 (2H, s), *δ*_H_ 4.09 (2H, dd, *J* = 12.0, 6.0 Hz), *δ*_H_ 3.64 (2H, t, *J* = 12.0 Hz), *δ*_H_ 3.49 (2H, d, *J* = 12.0 Hz), *δ*_H_ 2.37 (2H, t, *J* = 6.0 Hz), *δ*_H_ 2.12 (1H, m) and *δ*_H_ 2.09 (1H, m), *δ*_H_ 2.09 (2H, m), and *δ*_H_ 2.00 (2H, t, *J* = 12.0 Hz), alongside four methyl groups at *δ*_H_ 1.81 (3H, s), *δ*_H_ 1.66 (3H, s), *δ*_H_ 1.58 (3H, s), and *δ*_H_ 1.23 (3H, t, *J* = 6.0 Hz). Comprehensive analysis of ^13^C NMR and DEPT-135 spectral data identified 25 carbon resonances, corresponding to four methyls, eight methylenes, three methines (two olefinic carbons at *δ*_C_ 121.2 and 123.7, and an aromatic carbon at *δ*_C_ 97.8), and nine nonprotonated carbons (one ester carbonyl carbon at *δ*_C_ 173.3, one carbonyl at *δ*_C_ 169.2, and seven olefinic or aromatic carbons at *δ*_C_ 158.5, 150.7, 140.0, 132.4, 132.2, 121.2, and 118.4). A comparative analysis of 1D NMR data with those of isohericerin (**3**) demonstrated that **1** possessed an isoindolinone skeleton.

**Figure 2:**
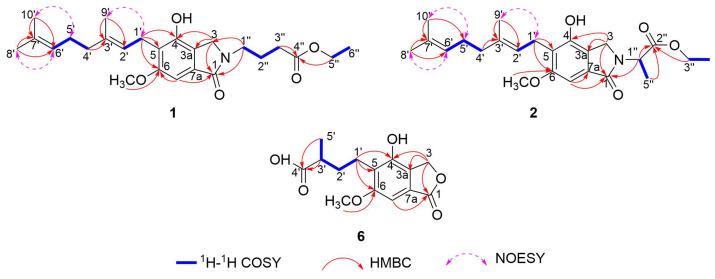
Key 2D NMR correlations of compounds **1**, **2**, and **6**.

The planar molecular structure was further confirmed through ^1^H−^1^H COSY and HMBC spectral analyses (Figure 2). The central isoindoline-1-one moiety was unambiguously corroborated by characteristic chemical shifts and pivotal HMBC cross-peaks. Specifically, diagnostic long-range HMBC correlations from H_2_-3 to C-3a, C-1, and C-4, along with those from H-7 to C-1 and C-6, securely anchored this core substructure. Meanwhile, the aliphatic chain attached at C-5 was identified by HMBC cross-peaks from H_3_-8′ to C-6′ and C-7′, from H_3_-10′ to C-6′ and C-7′, from H_2_-4′ to C-3′, from H_3_-9′ to C-2′ and C-3′, and from H_2_-1′ to C-4, C-5, and C-6, combined with the ^1^H−^1^H COSY spin systems of H_2_-4′/H_2_-5′/H-6′ and H_2_-1′/H-2′. The N-linked localization of the fatty acid chain was firmly established based on specific HMBC linkages from H_2_-1″ to C-1 and C-3, and from H_2_-5″ and H_2_-3″ to C-4″ coupled with ^1^H−^1^H COSY correlations of H_2_-1″/H_2_-2″/H_2_-3″ and H_2_-5″/H_3_-6″. Additionally, the incorporation of a methoxy substituent at C-6 was dictated by a pivotal HMBC linkage connecting the methoxy protons (*δ*_H_ 3.86) directly to C-6. Guided by the molecular formula of **1**, the notable downfield shift at C-4 (*δ*_C_ 150.7) served as diagnostic evidence for a hydroxyl substituent. Finally, the *E* configurations of Δ^2′,3′^ and Δ^6′,7′^ were evidenced by the ROESY correlations of H_2_-1′/H_3_-9′, H_2_-5′/H_3_-10′, and H-6′/H_3_-8′. Thus, the planar architecture of erinacerin X (**1**) was established as depicted in Figure 2.

**Figure 3:**
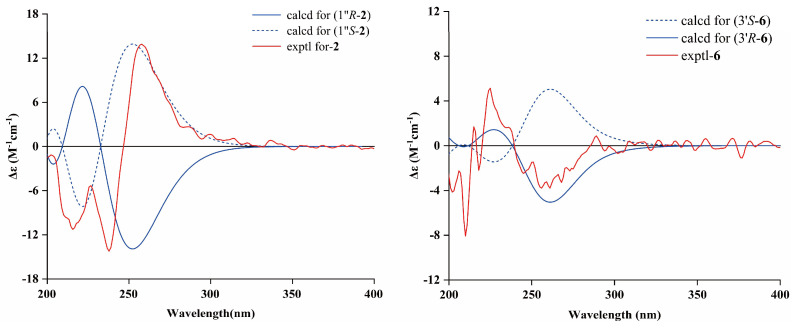
Experimental and calculated ECD spectra of **2** and **6**.

Integration of the HRESIMS and NMR data deduced the chemical constitution of erinacerin Y (**2**) to be C_24_H_33_NO_5_, which accounts for nine equivalents of unsaturation. The NMR spectra of compound **2** were highly similar to those of **1**, except for the presence of a C-1″−C-5″ fatty acid chain at the nitrogen atom instead of a C-1″−C-6″ chain (Table 1). This structural variation was validated by HMBC correlations from H-1″ to C-1, and from H-1″, H_3_-5″, and H_2_-3″ to C-2″, alongside the ^1^H−^1^H COSY spin systems of H-1″/H_3_-5″ and H_2_-3″/H_3_-4″. Detailed ROESY analysis (Figure 2) confirmed that **2** shared the same *E* configurations for Δ^2′,3′^ and Δ^6′,7′^ as **1**. To unambiguously assign the absolute stereochemistry of **2**, TDDFT calculations were executed via Gaussian 09 software. Theoretical ECD profiles for both epimers (1″*R*-**2** and 1″*S*-**2**) were generated by applying Δ*G*-dependent Boltzmann populations. Subsequent alignment of the acquired experimental ECD signature with the computational models revealed an excellent consistency with the 1″*S*-isomer (Figure 3), unequivocally establishing the absolute configuration at C-1″ as *S*.

Analysis of the [M-H]^−^ ion at *m*/*z* 279.0876 (calcd for C_14_H_15_O_6_, 279.0874) in the HRESIMS spectrum of erinacerin Z (**6**) disclosed a molecular formula of C_14_H_15_O_6_, corresponding to seven DBEs. The ^1^H NMR spectrum of **6** (Table 1) showed a singlet at *δ*_H_ 3.86 (3H, s) diagnostic for an aromatic methoxy substituent, a methyl signal at *δ*_H_ 1.26 (3H, d, *J* = 10.0 Hz), three methylenes at *δ*_H_ 5.25 (2H, s), *δ*_H_ 2.81 (1H, m) and 2.69 (1H, m), and *δ*_H_ 1.78 (2H, m), an aromatic singlet at *δ*_H_ 6.94 (1H, s), and a methine at *δ*_H_ 2.56 (1H, m). Combined ^13^C NMR and HSQC spectral analysis revealed 14 distinct carbon resonances attributed to a methyl group (*δ*_C_ 17.6), a methoxy carbon (*δ*_C_ 56.2), three methylenes (*δ*_C_ 68.5, 32.0, and 21.3), two methines including one aromatic (*δ*_C_ 98.1), and seven non-protonated carbons, which included two diagnostic carbonyl carbons (*δ*_C_ 182.3, 172.3), and five aromatic carbons (*δ*_C_ 159.6, 150.2, 127.7, 125.1, and 122.2). These data suggested that compound **6** possesses an isobenzofuranone skeleton.

The molecular structure of **6** was confirmed via long-range HMBC cross-peaks (Figure 2). The skeleton was corroborated by HMBC correlations linking H_2_-3 to C-4, C-3a, and C-1, and H-7 to C-1. Moreover, the ^1^H−^1^H COSY couplings of H_2_-1′/H_2_-2′/H-3′/H_3_-5′, combined with HMBC cross-peaks from H_3_-5′ and H-3′ to C-4′ and from H_2_-1′ to C-4, C-5, and C-6, confirmed the attachment of the alkyl chain at C-5. The specific C-4′ carbon resonance (*δ*_C_ 182.3) corroborated the existence of a free carboxylate functionality at the end of this aliphatic chain. Consistent with the structural features of **1**, the oxygenated functionalities in **6** were definitively mapped to C-6 (methoxy) and C-4 (hydroxyl). The 3’*R* absolute configuration was firmly assigned via comparative ECD computations (Figure 3).

Five previously reported isolates were characterized as isohericerin (**3**) [23], hericerin A (**4**) [24], N-dephenylethyl isohericerin (**5**) [23], corallocin A (**7**) [21], and 4-chloro-3,5-dimethoxybenzyl alcohol (**8**) [25] through spectral interpretation and comparison with published literature.

### 2.2. Results of Enzyme Activity Assay Enzyme Activity Assay

**Figure 4:**
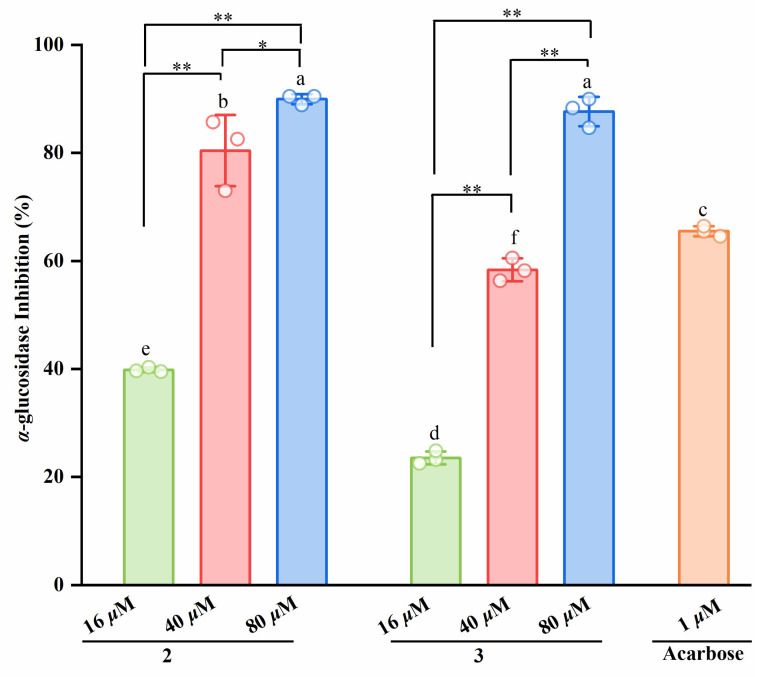
Inhibition effects of compounds **2** and **3** on *α*-glucosidase. Different letters (a–f) above bars indicate significant differences (*p* < 0.05) by Duncan’s multiple range test. * *p* < 0.05 and ** *p* < 0.01 versus the sample group.

All the isolated compounds (**1**–**8**) were evaluated in an in vitro *α*-glucosidase inhibition assay (Figure 4). As summarized in Table 2, the isoindolin-1-one derivatives **2** and **3** exhibited potent *α*-glucosidase inhibitory activity with IC_50_ values of 17.80 ± 1.03 μM and 19.50 ± 1.33 μM, respectively, substantially outperforming the other tested compounds. For a direct and meaningful comparison, the reference inhibitor acarbose was evaluated in parallel, displaying an IC_50_ value of 0.59 ± 0.13 μM. Subsequently, the active compounds (**2** and **3**) were used to further confirm the *α*-glucosidase inhibition assay at three different concentrations. Notably, at a concentration of 80 μM, compounds **2** and **3** suppressed *α*-glucosidase activity by approximately 90%, significantly surpassing the inhibition observed for the positive control acarbose when tested at 1 μM.

### 2.3. Molecular Docking Analysis Molecular Docking

**Figure 5:**
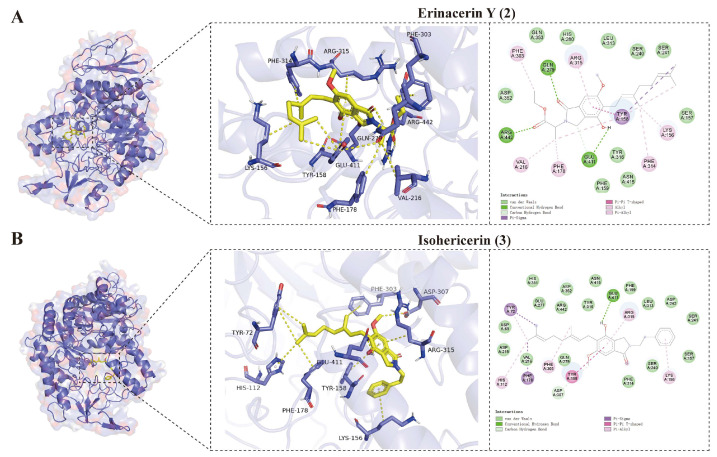
Interactions between compounds and *α*-glucosidase. (**A**) Interaction between erinacerin Y (**2**) and *α*-glucosidase. (**B**) Interaction between isohericerin (**3**) and *α*-glucosidase.

As an efficient tool for the evaluation of ligand–receptor binding affinity [26], molecular docking was performed for the eight isolated compounds and acarbose against the *α*-glucosidase receptor (PDB: 3A4A). The 3D/2D models illustrated key binding interactions within these compound–enzyme complexes (Figure 5 and Figure S31, and Table S1). Both **2** and **3** were well accommodated near the active pocket of *α*-glucosidase, yielding docking energies of −9.991 and −8.588 kcal/mol, respectively. To further evaluate the binding quality and mitigate the potential bias of molecular size on affinity, ligand efficiency (LE) values were calculated. Compound **2** exhibited an LE value of −0.33 kcal/mol/heavy atom. This macroscopic metric suggests that the structural scaffold of **2** is globally efficient, utilizing its heavy atoms compactly to achieve high binding affinity, while compound **3** showed a relatively lower efficiency with an LE of −0.27 kcal/mol/heavy atom. As shown in Figure 5A, compound **2** anchors near the active pocket via interactions with key residues, including Arg 442, Glu 411, Arg 315, Phe 314, Phe 303, Gln 279, Val 216, Phe 178, Tyr 158, and Lys 156. Specifically, Gln 279, Arg 442, and Glu 411 formed three critical hydrogen bonds with the C-1 carbonyl, the C-2″ carbonyl, and 4-OH group of **2**, respectively. Furthermore, the methyl substituents (at C-4″, C-5″, C-8′, and C-9′) and the phenyl ring established robust hydrophobic interactions with Phe 303, Val 216, Phe 178, Tyr 158, Lys 156, Phe 314, and Arg 315. Additionally, Tyr 158 engaged in π-stacking with the phenyl ring of **2** and formed a π-sigma interaction with the C-10′ methyl group. For compound **3** (Figure 5B), the docking model revealed that its 4-OH group forms a single hydrogen bond with Glu 411. Concurrently, hydrophobic interactions were observed with Arg 315, Lys 156, Phe 303, Phe 178, His 112, and Tyr 72 mediated by the isoindolin-1-one phenyl ring, the 3′-substituted phenyl ring, and the methyl groups at C-9′, C-8′, and C-10′. The phenyl ring of the isoindolin-1-one moiety also mediated a T-type π-π interaction with Tyr 158. Further stabilizing forces included van der Waals interactions between Asp 307 and the 6-OCH_3_ group, and a π-sigma interaction between the C-10′ methyl group and Tyr 72.

In comparison, the positive control acarbose primarily relies on its abundant hydroxyl groups to construct an extensive hydrogen bond network with key catalytic residues (e.g., Glu 411, Arg 315, Leu 313, Ser 311, Thr 310, and Asp 307) within the active site, further anchored by van der Waals interactions with Phe 303 (Figure S32). Notably, compound **2** shares critical interacting sites with acarbose, specifically Arg 315, Glu 411, and Phe 303, while compound **3** similarly interacts with Arg 315 and Phe 303. This overlapping interaction profile compellingly confirms that the compounds **2** and **3** binding adjacent to the core catalytic pocket. However, their microscopic binding strategies are fundamentally distinct. While acarbose relies heavily on widespread hydrophilic interactions, the isoindolin-1-one derivatives (**2** and **3**), despite forming fewer hydrogen bonds, leverage their unique scaffolds to establish dominant and highly robust hydrophobic networks and multiple π-mediated interactions. This differential binding mode effectively compensates for the reduced number of hydrogen bonds, thereby ensuring the exceptional binding energy of these natural products. Together, these structurally defined interactions provide a solid theoretical foundation for the inhibitory mechanisms of these natural product-derived agents.

### 2.4. MD Simulation Detail Analysis

**Figure 6:**
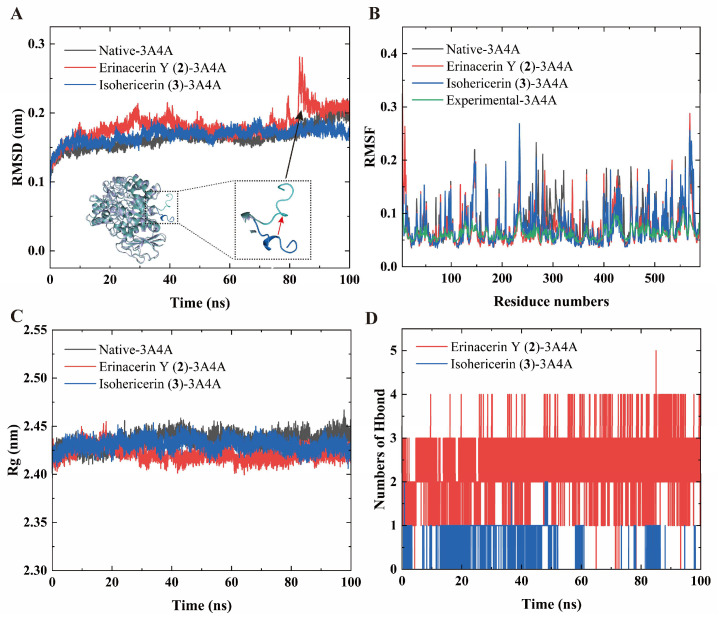
The results of molecular dynamics. (**A**) RMSD of the complexes. (**B**) RMSF of the complexes. (**C**) Rg and (**D**) Hydrogen bonding of **2** and **3** with 3A4A.

To validate the docking predictions and capture dynamic conformational changes, 100 ns all-atom MD simulations were performed on the free 3A4A enzyme and its complexes (**2**-3A4A and **3**-3A4A). The root-mean-square deviation (RMSD) serves as a primary indicator of structural stability for protein–ligand complexes, where lower values suggest a robust and stable interaction [26]. As depicted in Figure 6A, the RMSD trajectories of both **2**-3A4A and **3**-3A4A complexes reached a stable equilibrium after approximately 50 ns. The mean RMSD values for the latter half of the simulations (50–100 ns) were 0.18 ± 0.020 nm for **2**-3A4A and 0.17 ± 0.006 nm for **3**-3A4A. These values closely mirrored the native 3A4A trajectory (0.17 ± 0.010 nm), indicating that ligand binding maintained the overall structural stability of the enzyme. Minor fluctuations observed between 80 ns and 90 ns in the **2**-3A4A complex were attributable to the inherent flexibility of the N-terminal loop (residues 4–12, SSAHPTETP), which failed to disrupt the stable conformation of *α*-glucosidase (Figure 6A and Figure S33). The core RMSD analysis of the **2**-3A4A complex (excluding the flexible N-terminal residues) revealed highly stable dynamics throughout the simulation (Figure S34).

With the system stability confirmed to be stable, the root-mean-square fluctuation (RMSF) values were computed to examine the local flexibility of specific residues [27]. The overall RMSF patterns across all simulated systems were highly consistent with the experimental free state, validating the reliability of the simulations (Figure 6B). Compared to the free protein, the binding of ligands **2** and **3** significantly reduced the local flexibility of residues 140–160, 280–320, and 400–450, reflecting increased stability in the bound state. The compactness was corroborated by the radius of gyration (Rg) [26,28]. The Rg (Figure 6C) values of **2**-3A4A (2.419 ± 0.005 nm) and **3**-3A4A (2.430 ± 0.006 nm) were lower than that of the native free 3A4A (2.438 ± 0.006 nm), demonstrating that the binding of these ligands induces a more compact and stable protein conformation.

**Figure 7:**
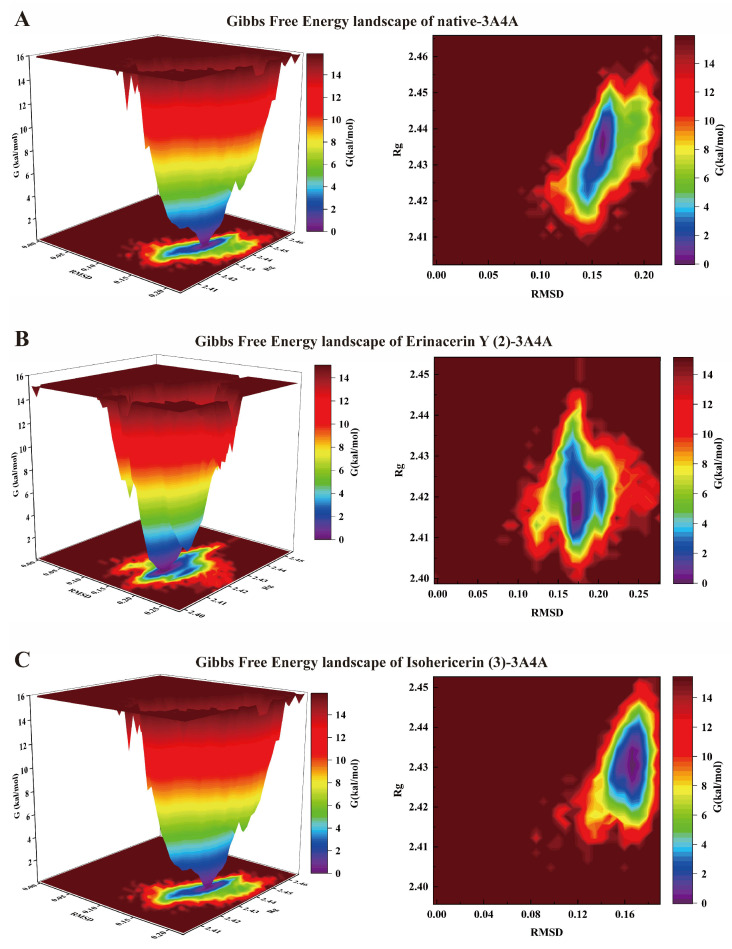
Gibbs free energy landscape for (**A**) **2**-3A4A, (**B**) **3**-3A4A, (**C**) Native-3A4A.

Hydrogen bonding is a critical determinant of binding specificity and strength. Time-evolution analysis over the 100 ns simulation (Figure 6D) revealed that compound **2** maintained an average of three stable intermolecular hydrogen bonds, whereas compound **3** sustained only one. Although the two compounds exhibit differing binding modes with the target protein, both form highly stable complexes with *α*-glucosidase. This dynamic observation perfectly corroborates the molecular docking results. Furthermore, the Gibbs free energy landscape [11,28] was constructed using RMSD and Rg as collective variables to characterize the conformational distribution sampled during the 100 ns simulation. As illustrated in Figure 7A–C, both the native system and the ligand-bound complexes predominantly sampled a single, dominant low-energy basin within this simulation window. Furthermore, within the evaluated simulation timescale, comprehensive trajectory analyses reveal that localized structural fluctuations (e.g., RMSDs originating from the flexible N-terminal loop) neither disrupted the global structural stability of the protein nor compromised the structural integrity of the ligand-binding pocket. These computational findings align well with our in vitro enzymatic inhibition data, collectively affirming the robust binding interactions.

**Figure 8:**
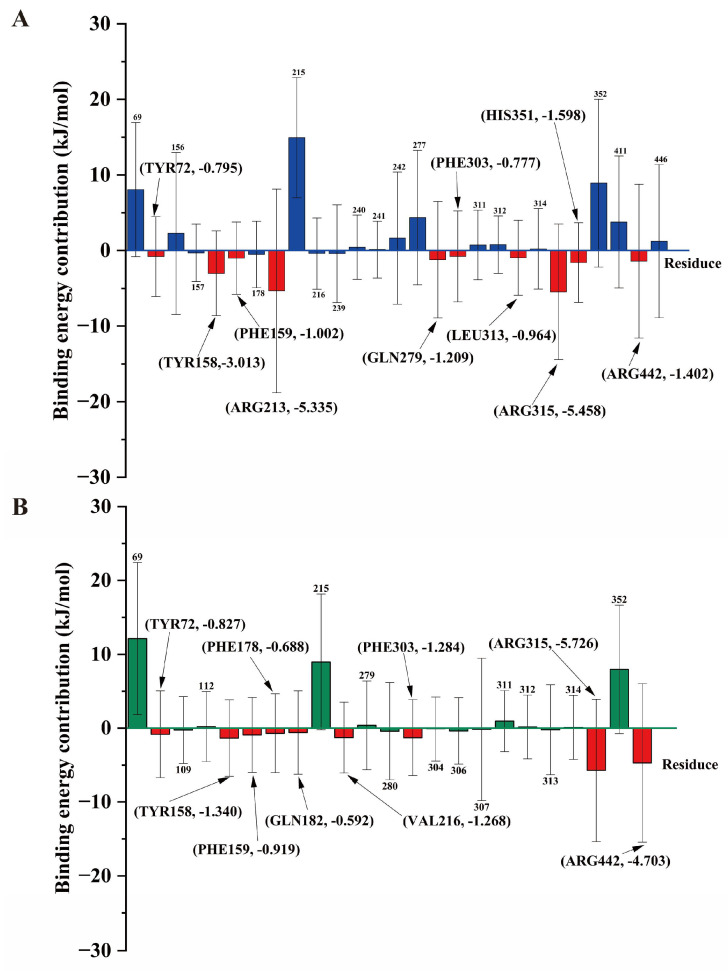
Residue energy contribution of 3A4A to the total binding energy in the **2**-3A4A (**A**) and **3**-3A4A (**B**) complexes.

To quantify the binding energies of these interactions, the MM-PBSA method was employed to estimate the affinities (Table 3). Consequently, the total free energy of binding was evaluated at −17.62 ± 6.12 kJ/mol for **2**-3A4A and −11.44 ± 5.40 kJ/mol for **3**-3A4A. Both electrostatic forces (Δ*E*_elec_) and van der Waals interactions (Δ*E*_vdW_) were identified as major contributors to complex stability. These findings align closely with recent investigations targeting *α*-glucosidase [27]. The significantly lower binding free energy of compound **2** indicates a stronger binding affinity, directly supporting its superior bioactivity. Per-residue binding energy decomposition was subsequently performed to highlight individual amino acid contributions (Figure 8). The stability of the **2**-3A4A complex (Figure 8A) was primarily driven by key residues including Arg 315 (−5.458 kJ/mol) and Arg 213 (−5.335 kJ/mol). Similarly, the **3**-3A4A complex (Figure 8B) was heavily stabilized by Arg 315 (−5.726 kJ/mol). These shared interaction hotspots highlight their critical roles in *α*-glucosidase inhibition.

### 2.5. Validation of Specific Binding Affinity by SPR

The binding affinity of compounds **2** and **3** for α-glucosidase were validated using SPR assays. As determined by this assay, the two compounds bound to *α*-glucosidase with significantly different affinities, having K_D_ values of 24.91 μM and 89.84 μM, respectively (Figure S35). This approximately 3.6-fold stronger binding affinity of **2** over **3** suggests that **2** forms a kinetically more stable complex with the enzyme. Crucially, these experimental binding trends align well with the docking energies and MD free-energy calculations, providing a robust and validation of their mechanism of action as potent *α*-glucosidase inhibitors.

## 3. Materials and Methods

### 3.1. General Experimental Procedures

Optical rotation values were determined employing an Autopol VI polarimeter (Rudolph Instruments, NJ, USA). Additionally, FT-IR and UV-Vis spectra were acquired on a Thermo Scientific NICOLET iS5 spectrometer (Thermo Fisher Scientific, Madison, WI, USA) and a Varian Cary 100 spectrophotometer (Varian, Inc., Palo Alto, CA, USA), respectively. A Thermo Q Exactive HF Orbitrap-MS system (Thermo Fisher Scientific, Bremen, Germany) was deployed for HRESIMS measurements, whereas the acquisition of electronic circular dichroism (ECD) data was achieved with a Jasco J-810 spectropolarimeter (JASCO Corporation, Hachioji, Tokyo, Japan). All NMR spectroscopic measurements were performed on a 500 MHz Bruker AV spectrometer (Bruker BioSpin, Billerica, MA, USA). Samples were dissolved in CDCl_3_, which also provided the references (*δ*_H_ 7.26, *δ*_C_ 77.16). Separations via preparative medium-pressure liquid chromatography (MPLC) were conducted utilizing a Chuangxintongheng LC 3000 instrument (Beijing Chuangxintongheng Science & Technology Co., Ltd., Beijing, China), which was fitted with an Agilent Zorbax Eclipse Plus C18 PrepHT column (21.2 × 250 mm, 5 μm). For column chromatographic fractionations, normal-phase silica gel (200–300 mesh; Yantai, China) and reversed-phase ODS C18 (15 μm; Santai Technologies, Inc., Montreal, QC, Canada) were deployed as the stationary phases. Analytical and preparative TLC profiling was conducted using silica gel FSGF254 plates, with HPLC analyses relying on a YMC-Pack Pro C18 (250 × 10 mm, 5 μm). All solvents utilized for HPLC separations were of HPLC-grade, whereas those used for extraction and MPLC were of analytical grade.

### 3.2. Fungus Material

*Hericium erinaceus* was initially isolated from naturally grown fruiting bodies, and its species identification was verified by Sangon Biotech (Shanghai, China) Co., Ltd. via molecular sequencing. The mushroom specimen was deposited at the National Edible Fungi Germplasm Resources Collection Center (Shanghai, China) under the strain collection number 7080.

### 3.3. Fermentation, Extraction, and Isolation

The *H. erinaceus* strain was initially maintained on PDA at 25 °C. An aqueous liquid medium consisting of anhydrous glucose (30 g/L), standard glucose (12 g/L), yeast powder (3 g/L), KH_2_PO_4_ (2.0 g/L), and MgSO_4_·7H_2_O (2.0 g/L) was prepared for stages. Volumes of 100 mL of this broth in 250 mL flasks were inoculated with spores and subjected to 7 days shake (150 rpm) at 26 °C. Following this, 200 mL of the aforementioned medium in 500 mL flasks was seeded with a 10% aliquot of the first-generation culture, followed by an additional 7 days incubation under identical conditions. The subsequent second-generation cultures were transferred to 2 L fermentation boxes and incubated statically at 26°C in the dark for three weeks. The upper mycelia were harvested, freeze-dried, and extracted with a 95% (*v*/*v*) aqueous ethanol solution at room temperature. The resulting brown extract (113.7 g) was partitioned three times with petroleum ether and EtOAc (3× volume each). After removing the organic solvent under reduced pressure, 38.2 g of the EtOAc fraction was obtained.

The crude EtOAc fraction (38.2 g) was subjected to MPLC separation on a YMC Gel column, eluted with a linear gradient of MeCN: H_2_O (20:80 to 100:0, *v*/*v*) to give fifteen fractions (Frs. 1−15). Fr. 7 was separated by reversed-phase ODS MPLC (80−30% H_2_O + 0.01% HCOOH/MeCN, 60 min, flow rate 15 mL/min, UV detected at 210 nm) to afford two subfractions (Frs. 7.1 and 7.2). These were further purified via preparative HPLC, eluted with 80% H_2_O + 0.01% HCOOH/MeCN and 79% H_2_O + 0.01% HCOOH/MeCN, to yield **8** (12.9 mg, *t*_R_ = 43.6 min) and **6** (4.8 mg, *t*_R_ = 61.3 min), respectively. Fr. 10 was purified by preparative HPLC (63−58% H_2_O + 0.01% HCOOH/MeCN, 45 min) to give Fr. 10.1, which was further refined (63−60% H_2_O + 0.01% HCOOH/MeCN) to yield **7** (13.8 mg, *t*_R_ = 37.1 min). Subfraction 11.1 was generated by processing Fr. 11 through HPLC, utilizing a gradient of 58–51% acidified aqueous solution (0.01% HCOOH in H_2_O) against MeCN over 40 min (flow rate: 15 mL/min; UV detection: 220 nm). Subsequent purification of this intermediate over a gel chromatography column with isocratic MeOH elution furnished compound **4** (17.5 mg). Fr. 12 underwent HPLC fractionation (MeCN with 55–38% aqueous 0.01% HCOOH, 50 min; flow rate: 20 mL/min; UV: 210 nm), which successfully isolated compounds **1** (14.1 mg, *t*_R_ = 40.9 min) and **2** (8.9 mg, *t*_R_ = 45.3 min) in addition to Fr. 12.1. The latter was then subjected to recrystallization to generate compound **5** (26.8 mg). Similarly, compound **3** (33.0 mg) was crystallized from Fr. 15 following the aforementioned procedure.

Erinacerin X (**1**): light-yellow oil; [*α*${]}_{D}^{25}$ −1.00 (*c* 0.200, MeOH); UV (MeOH) λ_max_ (log *ε*) 293 (3.49), 258 (3.85), 213 (4.39) nm; IR (ATR) *v_max_* 3415, 2995, 2945, 2833, 1733, 1668, 1473, 1033 cm^−1^; ^1^H and ^13^C NMR data are provided in Table 1; HRESIMS *m*/*z* 452.2404 [M + Na]^+^ (C_25_H_35_NO_5_Na, calcd for 452.2407).

Erinacerin Y (**2**): light-yellow oil; [*α*${]}_{D}^{25}$ + 2.40 (*c* 0.200, MeOH); UV (MeOH) λ_max_ (log *ε*) 260 (3.96), 213 (4.47) nm; IR (ATR) *v_max_* 3349, 3009, 2949, 2830, 1745, 1666, 1471, 1031 cm^−1^; ^1^H and ^13^C NMR data are provided in Table 1; HRESIMS *m*/*z* 438.2240 [M + Na]^+^ (C_24_H_33_NO_5_Na, calcd for 438.2251).

Erinacerin Z (**6**): colorless amorphous powder; [*α*${]}_{D}^{25}$ −2.00 (*c* 0.200, MeOH); UV (MeOH) λ_max_ (log *ε*) 303 (3.53), 260 (3.96), 213 (4,53) nm; IR (ATR) *v_max_* 3363, 3001, 2945, 2832, 1737, 1471,1031 cm^−1^; ^1^H and ^13^C NMR data are provided in Table 1; HRESIMS *m*/*z* 279.0876 [M-H]^−^ (C_14_H_15_O_6_, calcd for 279.0874).

### 3.4. TDDFT ECD Calculation

A systematic random search in Sybyl-X 2.0 (employing the MMFF94S force field with an upper energy limit of 5.0 kcal/mol cutoff) was utilized to generate the preliminary conformational ensembles [29]. Following this step, the viable conformers for **2** and **6** were further optimized at the DFT/B3LYP/6-311+G(d,p) level in a methanol solvent model, utilizing Gaussian 09 package. ECD spectral curves were subsequently generated by applying a Gaussian-type function overlapping approach. The overall theoretical ECD curves for compounds **2** and **6** were generated by averaging the calculated spectra of all stable conformers, utilizing Boltzmann weighting derived from their respective relative Gibbs free energies (∆*G*).

### 3.5. Molecular Docking

For the computational simulations, the three-dimensional crystal coordinates of the *α*-glucosidase target (PDB ID: 3A4A) were retrieved directly from the Protein Data Bank. Chem3D 22.0.0 was deployed to build the three-dimensional structures of the novel compounds (**1**, **2**, and **6**). Meanwhile, the NCBI PubChem database served as the structural source for the remaining known ones (**3**, **4**, **5**, **7**, and **8**). Autodock Tools (ADT) 1.5.7 was employed to assign rotatable bonds within the enzyme and ligands, after which molecular docking simulations were performed and optimized [30,31]. The affinity of the receptor-ligand interactions was assessed based on molecular docking scores.

### 3.6. MD Simulation

MD simulations were executed using GROMACS version 2024.2 [32]. The AMBER14SB_PARMBSC1.FF all-atom force field, combined with the TIP3P explicit solvent water model, were applied to the **2**-3A4A, **3**-3A4A, and free 3A4A molecular docking complexes [33,34]. The topologies and parameters for the small-molecule ligands were generated utilizing the General AMBER Force Field (GAFF). Unconstrained simulations were performed within a cubic box (minimum edge distance of 1.0 nm) and conducted at 298.15 K, 1 atm pressure, and physiological salt concentration. Energy minimization was first performed with a force tolerance of 100.0 kJ mol^−1^ nm^−1^, followed by a 300 ps position-restrained dynamics simulation on the protein atoms using a 1 fs time step. Unrestrained production runs spanned a 100 ns timeframe, integrating the equations of motion with a 2 fs time step. Long-range electrostatic forces were handled via the Particle Mesh Ewald (PME) algorithm, while a uniform 1.0 nm real-space cutoff was applied to both Coulombic and short-range van der Waals interactions. Pressure was regulated using the Parrinello-Rahman barostat [35,36]. Post-simulation trajectory analyses were executed utilizing standard GROMACS utility programs. Binding free energy calculations and per-residue binding energy decompositions were performed using the gmx-based MMPBSA program [37].

### 3.7. Enzyme Activity Assay

The enzyme inhibition assay was conducted based on previously described methods [20,38]. The *α*-glucosidase inhibitory assay was performed in 96-well plates. 20 μL aliquots of either the test isolates (yielding final assay concentrations of 8, 16, 40, 80, and 160 μM) or the positive control acarbose (final concentrations of 0.03 μM, 0.1 μM, 0.3 μM, 1 μM, 3 μM, and 10 μM) were mixed with 180 μL of PBS and 25 μL of the enzyme solution. This baseline mixture underwent a 10 min pre-incubation period at 26 °C. Catalysis was triggered by the addition of 25 μL of 23.2 mM 4-nitrophenyl-*α*-D-glucopyranoside, followed by a final incubation stage at 37 °C lasting 15 min. The quantification of the enzymatic product was achieved by monitoring the absorbance at 405 nm utilizing a microplate reader. For the blank group, 20 μL DMSO was added instead of the compound; the positive control group utilized acarbose (1 μM); and for the background group, 25 μL PBS was substituted for *α*-glucosidase. The extent of *α*-glucosidase inhibition (%) was determined based on the relationship defined below:

$$\frac{\left[\left(OD_{control}-OD_{control\;blank}\right)-\left(OD_{sample}-OD_{sample\;blank}\right)\right]}{OD_{control}-OD_{control\;blank}}\times 100\%.$$

IBM SPSS Statistics 20 was utilized to determine the IC_50_ values via non-linear regression.

### 3.8. Assessment of the Binding Affinity of Compounds 2 and 3 for α-Glucosidase

A Biacore X100 system (GE Healthcare Bioscience AB, Uppsala, Sweden) was used to analyze the binding interactions between the tested compounds and the protein at 25 °C. Immobilization of *α*-glucosidase was achieved on a CM5 research grade sensor chip via primary amine linkage, facilitated by the reagents from an Amine Coupling Kit (Cytiva, Marlborough, MA, USA). The final immobilization level of *α*-glucosidase was approximately 11,309 response units (RU). To maintain a constant physiological environment during the binding assays, a PBS-P solution (10 mM) containing 0.05% surfactant P20, 2.7 mM KCl, and 137 mM NaCl was employed as the standard running buffer. Test compounds at various concentration gradients were injected as analytes and flowed over the chip surface. Binding assays were performed using the running buffer containing the analytes at appropriate concentrations. The process was conducted at a flow rate of 30 μL/min, with a 60 s of contact and a 120 s of dissociation time, followed by chip regeneration with running buffer in combination with 50% DMSO. The dissociation constant (K_D_) was derived from nonlinear curve fitting analysis based on kinetic and steady-state affinity models using the Biacore X100 evaluation software (version 2.0) [39,40].

## 4. Conclusions

In the present study, three new compounds, erinacerins X−Z (**1**, **2**, and **6**), were isolated and identified from the mycelia-associated *H. erinaceus*, along with five known ones (**3**–**5**, **7**, and **8**). Subsequent in vitro evaluations revealed that the isoindolin-1-one derivatives, compounds **2** and **3**, exhibit notable activity as naturally derived *α*-glucosidase inhibitors. To elucidate their specific mechanisms of action, molecular docking and MD simulations were conducted. These computational analyses demonstrated that compounds **2** and **3** bind stably near the active pocket of *α*-glucosidase, primarily driven by van der Waals interactions and hydrogen bonds. Furthermore, the simulations indicated a stronger binding free energy for the **2**-3A4A complex compared to the **3**-3A4A complex, underscoring the crucial roles of electrostatic and van der Waals interactions forces in stabilizing the complexes. This computational trend was strongly corroborated by SPR analysis, which experimentally confirmed the higher binding affinity of compound **2** (K_D_ = 24.91 μM) over **3** (K_D_ = 89.84 μM). Together, these biophysical and in silico results perfectly align with the initial inhibitory activity data. Ultimately, these findings highlight that mycelia-associated *Hericium* fungi represent a valuable resource library for the discovery of novel bioactive products with potential medicinal applications.

## Figures and Tables

**Table 1:** ^1^H NMR (500 MHz) and ^13^C NMR (125 MHz) spectroscopic data for compounds **1**, **2**, and **6** in CDCl_3_.

	1		2		6	
No.	*δ_H_*	*δ_C_*	*δ_H_*	*δ_C_*	*δ_H_*	*δ_C_*
1		169.2, C		169.2, C		172.3, C
3	4.28 s	47.7, CH_2_	4.30 d (18.0)	44.5, CH_2_	5.25 s	68.5, CH_2_
			4.44 d (12.0)			
3a		132.2, C		131.7, C		127.7, C
4		150.7, C		150.6, C		150.2, C
5		118.4, C		118.6, C		122.2, C
6		158.5, C		158.5, C		159.6, C
7	6.97 s	97.8, CH	6.97 s	97.9, CH	6.94 s	98.1, CH
7a		121.2, C		121.9, C		125.1, C
1′	3.49 d (12.0)	22.9, CH_2_	3.49 d (6.0)	22.9, CH_2_	2.81 m	21.3, CH_2_
					2.69 m	
2′	5.25 t (12.0)	121.2, CH	5.25 t (6.0)	121.1, CH	1.78 m	32.0, CH_2_
3′		140.0, C		140.0, C	2.56 m	38.5, CH
4′	2.09 m	39.8, CH_2_	2.09 m	39.8, CH_2_		182.3, C
5′	2.12 m	26.4, CH_2_	2.12 m	26.4, CH_2_	1.26 d (10.0)	17.6, CH_3_
	2.09 m		2.09 m			
6′	5.03 t (6.0)	123.7, CH	5.04 t (6.0)	123.7, CH		
7′		132.4, C		132.5, C		
8′	1.66 s	25.9, CH_3_	1.68 s	25.9, CH_3_		
9′	1.81 s	16.3, CH_3_	1.83 s	16.4, CH_3_		
10′	1.58 s	17.9, CH_3_	1.60 s	17.9, CH_3_		
1″	3.64 t (12.0)	42.0, CH_2_	5.15 m	49.5, CH		
2″	2.00 t (12.0)	23.9, CH_2_		172.0, C		
3″	2.37 t (6.0)	31.6, CH_2_	4.18 dd (6.0, 12.0)	61.5, CH_2_		
4″		173.3, C	1.26 t (6.0)	14.3, CH_3_		
5″	4.09 dd (6.0, 12.0)	60.7, CH_2_	1.56 d (12.0)	16.0, CH_3_		
6″	1.23 t (6.0)	14.3, CH_3_				
6-OCH_3_	3.86 s	56.3, CH_3_	3.86 s	56.3, CH_3_	3.86 s	56.2, CH_3_

**Table 2:** *α*-Glucosidase inhibition assay of compounds **1**−**8** and acarbose (IC_50_ values in μM).

Comp.	IC_50_ (μM)
**1**	40.08 ± 0.65
**2**	17.80 ± 1.03
**3**	19.50 ± 1.33
**4**	-
**5**	31.29 ± 1.89
**6**	-
**7**	76.49 ± 3.2
**8**	-
**Acarbose**	0.59 ± 0.13

“-“: No *α*-glucosidase inhibition activity.

**Table 3:** Energy Components of **2**-3A4A and **3**-3A4A Complexes (kJ/mol).

Energy Components	Erinacerin Y (2)	Isohericerin (3)
Δ_vdW_	−65.63 ± 2.75	−53.92 ± 2.62
Δ*E*_elec_	−32.63 ± 5.00	−8.90 ± 2.72
Δ*E*_PB_	85.79 ± 6.12	56.75 ± 6.18
Δ*E*_nonpolar_	−5.61 ± 0.08	−5.37 ± 0.23
Δ*G*_gas_	−97.25 ± 4.81	−62.82 ± 4.06
Δ*G*_solvation_	80.17 ± 6.12	51.38 ± 6.11
Δ*G*_Bind_	−17.62 ± 6.12	−11.44 ± 5.40

## Data Availability

The original contributions presented in this study are included in the article/Supplementary Material. Further inquiries can be directed to the corresponding authors.

## Supplementary Materials

The following supporting information can be downloaded at: https://www.mdpi.com/article/10.3390/molecules31101605/s1, Figure S1: HRESIMS spectrum of compound **1**; Figure S2: IR spectrum of compound **1**; Figure S3: UV spectrum of compound **1**; Figure S4: ^1^H NMR spectrum of compound **1** in CDCl_3_; Figure S5: ^13^C NMR spectrum of compound **1** in CDCl_3_; Figure S6: DEPT 135 spectrum of compound **1** in CDCl_3_; Figure S7: ^1^H−^1^H NMR spectrum of compound **1** in CDCl_3_; Figure S8: HSQC spectrum of compound **1** in CDCl_3_; Figure S9: HMBC spectrum of compound **1** in CDCl_3_; Figure S10: NOESY spectrum of compound **1** in CDCl_3_; Figure S11: HRESIMS spectrum of compound **2**; Figure S12: IR spectrum of compound **2**; Figure S13: UV spectrum of compound **2**; Figure S14: ^1^H NMR spectrum of compound **2** in CDCl_3_; Figure S15: ^13^C NMR spectrum of compound **2** in CDCl_3_; Figure S16: DEPT 135 spectrum of compound **2** in CDCl_3_; Figure S17: ^1^H−^1^H NMR spectrum of compound **2** in CDCl_3_; Figure S18: HSQC spectrum of compound **2** in CDCl_3_; Figure S19: HMBC spectrum of compound **2** in CDCl_3_; Figure S20: NOESY spectrum of compound **2** in CDCl_3_; Figure S21: HRESIMS spectrum of compound **6**; Figure S22: IR spectrum of compound **6**; Figure S23: UV spectrum of compound **6**; Figure S24: ^1^H NMR spectrum of compound **6** in CDCl_3_; Figure S25: ^13^C NMR spectrum of compound **6** in CDCl_3_; Figure S26: DEPT 135 spectrum of compound **6** in CDCl_3_; Figure S27: ^1^H−^1^H NMR spectrum of compound **6** in CDCl_3_; Figure S28: HSQC spectrum of compound **6** in CDCl_3_; Figure S29: HMBC spectrum of compound **6** in CDCl_3_; Figure S30: NOESY spectrum of compound **6** in CDCl_3_; Figure S31: Molecular docking between compounds (**1**–**8** and acarbose) and *α*-glucosidase; Figure S32: Interactions between acarbose and *α*-glucosidase; Figure S33: Conformational change in the N-Terminal Residues 4-12 (8261st in purple and 8331st in blue; Figure S34: Adjusted RMSD of the **2**-3A4A complex (excluding flexible N-terminal residues 4–12, SSAHPTETP); Figure S35: Specific binding affinity by SPR; Table S1: The molecular docking results for 3A4A with compounds **1**–**8** and acarbose.
