# Supplementary material for: Discovery of Natural α-Glucosidase Inhibitors from Hericium erinaceus Through Integrated Isolation, Structural Characterization, In Vitro Evaluation, and Molecular Dynamics Simulations
Source: Molecules. 2026 May 11;31(10):1605. doi: 10.3390/molecules31101605 (PMC13209448; doi:10.3390/molecules31101605)
Supplement: Supplementary file 1 [file molecules-31-01605-s001.zip › molecules-4300653-supplementary.pdf]

# Supporting Information

# Discovery of Natural *α*-Glucosidase Inhibitors from *Hericium erinaceus* Through Integrated Isolation, Structural Characterization, In Vitro Evaluation, and Molecular Dynamics Simulations

**Xianxian Miao [1,†], Xiangming Kong [2,†], Xiaodong Shang [1], Yan Yang [1], Wei Han [2,*], Jingsong Zhang [1,*] and Na Feng [1,*]**

[1] Institute of Edible Fungi, Shanghai Academy of Agricultural Sciences, Shanghai 201403, China; mrx@saas.sh.cn (X.M.); shangxiaodong@saas.sh.cn (X.S.); yangyan@saas.sh.cn (Y.Y.)

[2] Shanghai Key Laboratory of New Drug Design, Engineering Research Center of Pharmaceutical Process Chemistry, Ministry of Education, School of Pharmacy, East China University of Science and Technology, Shanghai 200237, China; 20240505@saas.sh.cn

* Correspondence: whan@ecust.edu.cn (W.H.); syja16@saas.sh.cn (J.Z.); fengna@saas.sh.cn (N.F.)

† These authors contributed equally to this work.

## Contents

Figure S1. HRESIMS spectrum of compound **1**. ........ 3
Figure S2. IR spectrum of compound **1**........ 4
Figure S3. UV spectrum of compound **1**. ........ 4
Figure S4. $^1$H NMR spectrum of compound **1** in $CDCl_3$........ 5
Figure S5. $^{13}$C NMR spectrum of compound **1** in $CDCl_3$........ 5
Figure S6. DEPT135 spectrum of compound **1** in $CDCl_3$. ........ 6
Figure S7.$^1$H-$^1$H NMR spectrum of compound **1** in $CDCl_3$. ........ 6
Figure S8. HSQC spectrum of compound **1** in $CDCl_3$. ........ 7
Figure S9. HMBC spectrum of compound **1** in $CDCl_3$. ........ 7
Figure S10. NOESY spectrum of compound **1** in $CDCl_3$........ 8
Figure S11. HRESIMS spectrum of compound **2**. ........ 8
Figure S12. IR spectrum of compound **2**........ 9
Figure S13. UV spectrum of compound **2**. ........ 10
Figure S14. $^1$H NMR spectrum of compound **2** in $CDCl_3$........ 11
Figure S15. $^{13}$C NMR spectrum of compound **2** in $CDCl_3$........ 11
Figure S16. DEPT135 spectrum of compound **2** in $CDCl_3$. ........ 12
Figure S17.$^1$H-$^1$H NMR spectrum of compound **2** in $CDCl_3$. ........ 12
Figure S18. HSQC spectrum of compound **2** in $CDCl_3$. ........ 13
Figure S19. HMBC spectrum of compound **2** in $CDCl_3$. ........ 13
Figure S20. NOESY spectrum of compound **2** in $CDCl_3$........ 14
Figure S21. HRESIMS spectrum of compound **6**. ........ 14
Figure S22. IR spectrum of compound **6**........ 15
Figure S23. UV spectrum of compound **6**. ........ 16
Figure S24. $^1$H NMR spectrum of compound **6** in $CDCl_3$........ 17
Figure S25. $^{13}$C NMR spectrum of compound **6** in $CDCl_3$........ 17
Figure S26. DEPT135 spectrum of compound **6** in $CDCl_3$. ........ 18
Figure S27. HSQC spectrum of compound **6** in $CDCl_3$. ........ 18
Figure S28. $^1$H-$^1$H NMR spectrum of compound **6** in $CDCl_3$. ........ 19
Figure S29. HMBC spectrum of compound **6** in $CDCl_3$. ........ 19
Figure S30. NOESY spectrum of compound **6** in $CDCl_3$........ 20

Figure S31. Molecular docking between compounds (**1**−**8** and acarbose) and *α*-glucosidase. 21

Figure S32. Interactions between acarbose and *α*-glucosidase. 22

Figure S33. Conformational change of the N-Terminal Residues 4-12 (8261st in purple and 8331st in blue). 22

Figure S34. Adjusted RMSD of the **2**-3A4A complex (excluding flexible N-terminal residues 4–12, SSAHPTETP). 23

Figure S35 Specific binding affinity by SPR. 24

Table S1. The molecular docking results for 3A4A with compounds**1**−**8** and acarbose 24

Figure S1. HRESIMS spectrum of compound **1**.

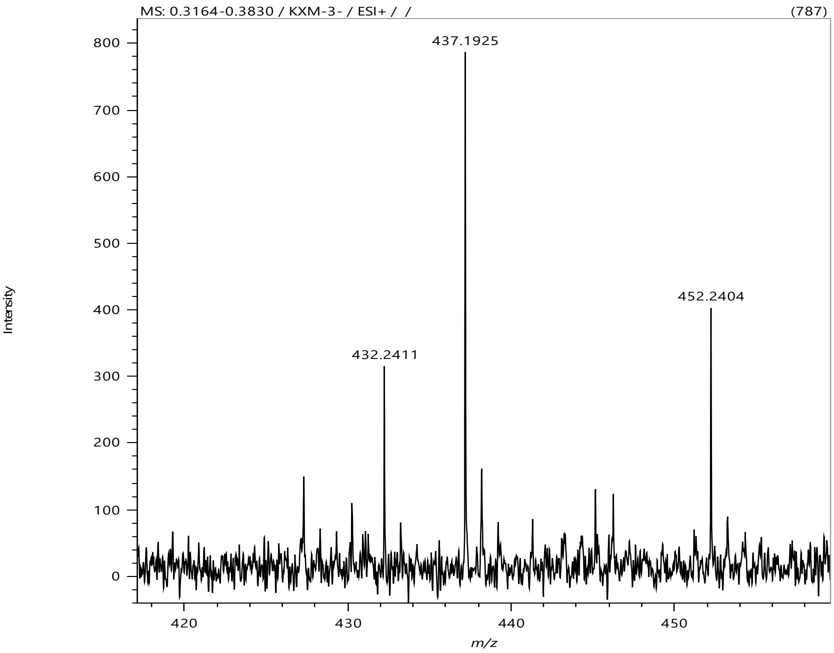


| Mass | Intensity | Formula | Calculated Mass | Mass Difference [ppm] | DBE |
|---|---|---|---|---|---|
| 452.24043 | 402.40 | C25 H35 N O5 Na | 452.24074 | -0.69 | 8.5 |

Figure S2. IR spectrum of compound **1**.

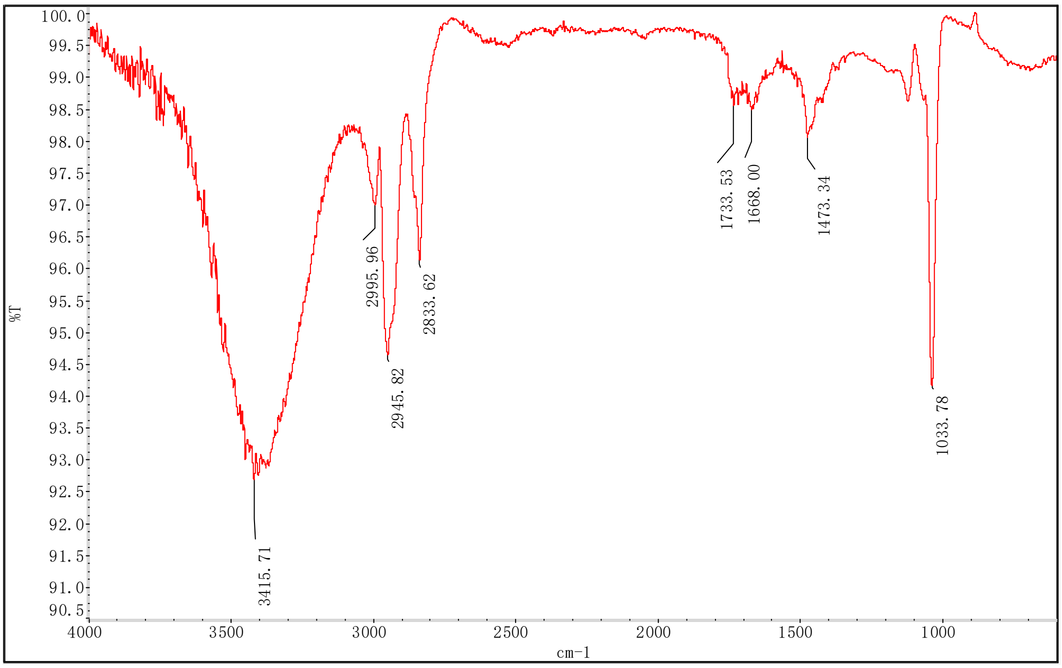


Figure S3. UV spectrum of compound **1**.

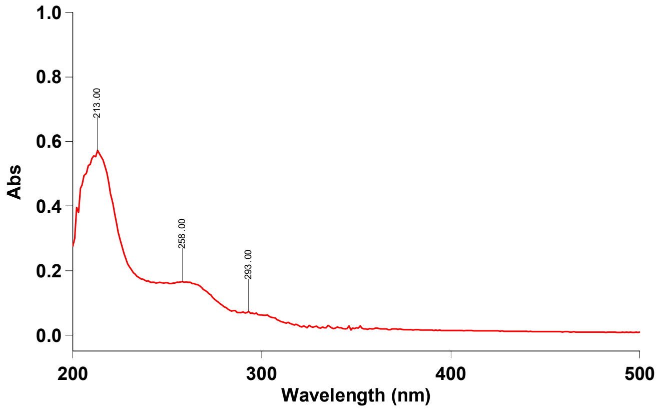


Scan Analysis Report

Report Time :   !"    05 #$    02:31:44 PM 2024
Method:
Batch: E:\YZR\20240105\KXM-3.DSW
Software version: 3.00(339)
Operator:

Sample Name: KXM-3 0.01mg-mL
Collection Time    2024-1-5 14:30:39

Peak Table
Peak Style    Peaks
Peak Threshold    0.0040
Range    500.00nm to 200.00nm

| Wavelength (nm) | Abs |
| --- | --- |
| 293.00 | 0.072 |
| 258.00 | 0.166 |
| 213.00 | 0.572 |

Figure S4. $^{1}$H NMR spectrum of compound **1** in CDCl3.

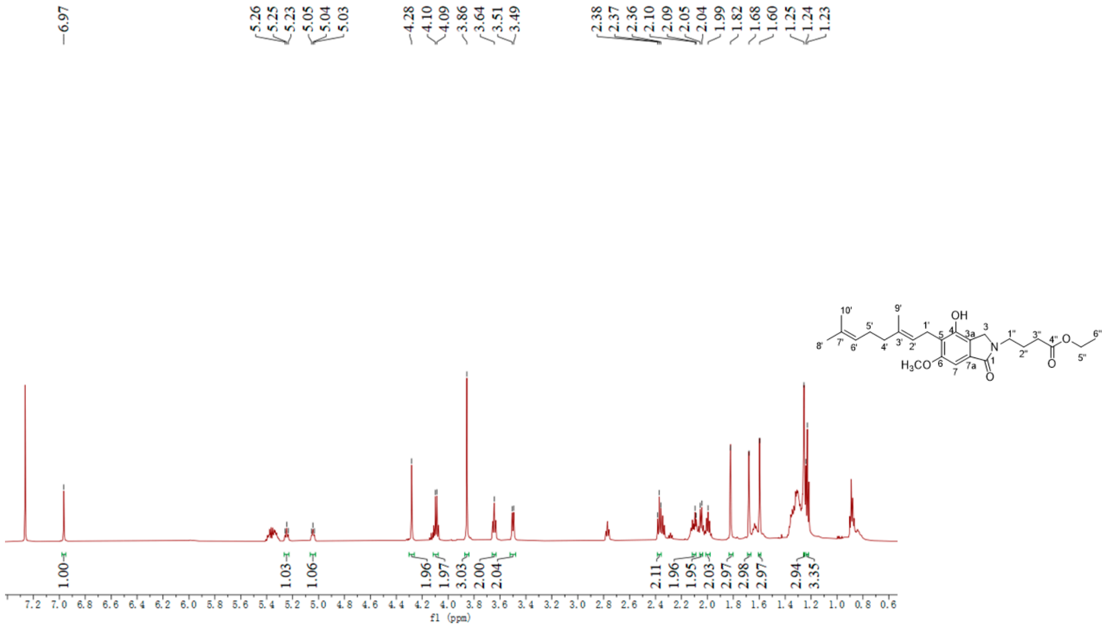


Figure S5. $^{13}$C NMR spectrum of compound **1** in CDCl3.

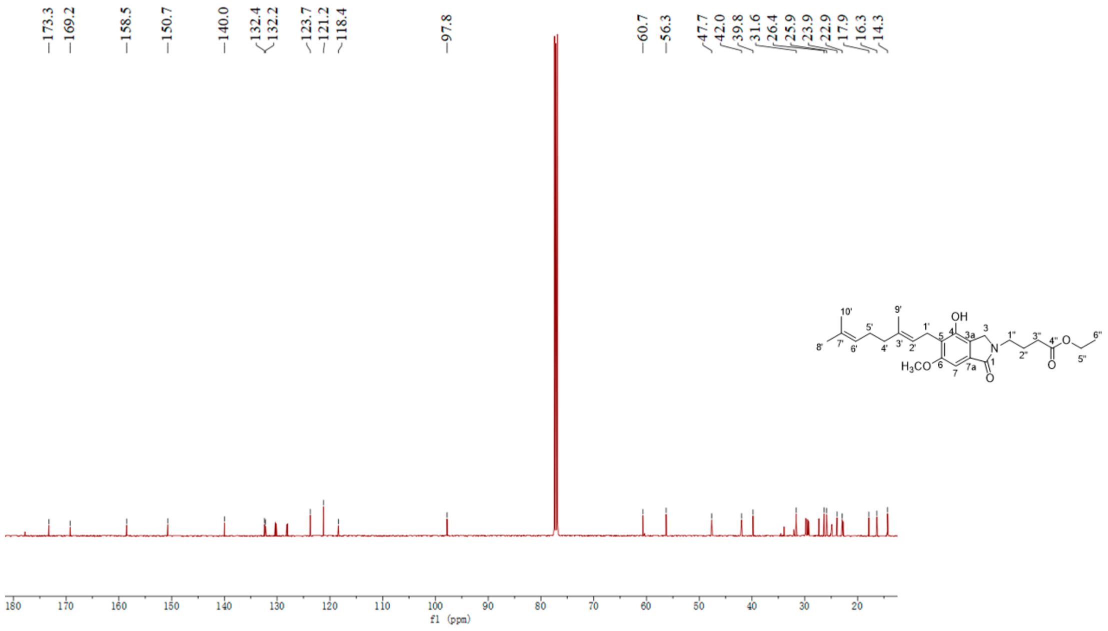

Figure S6. DEPT135 spectrum of compound **1** in $CDCl_3$.

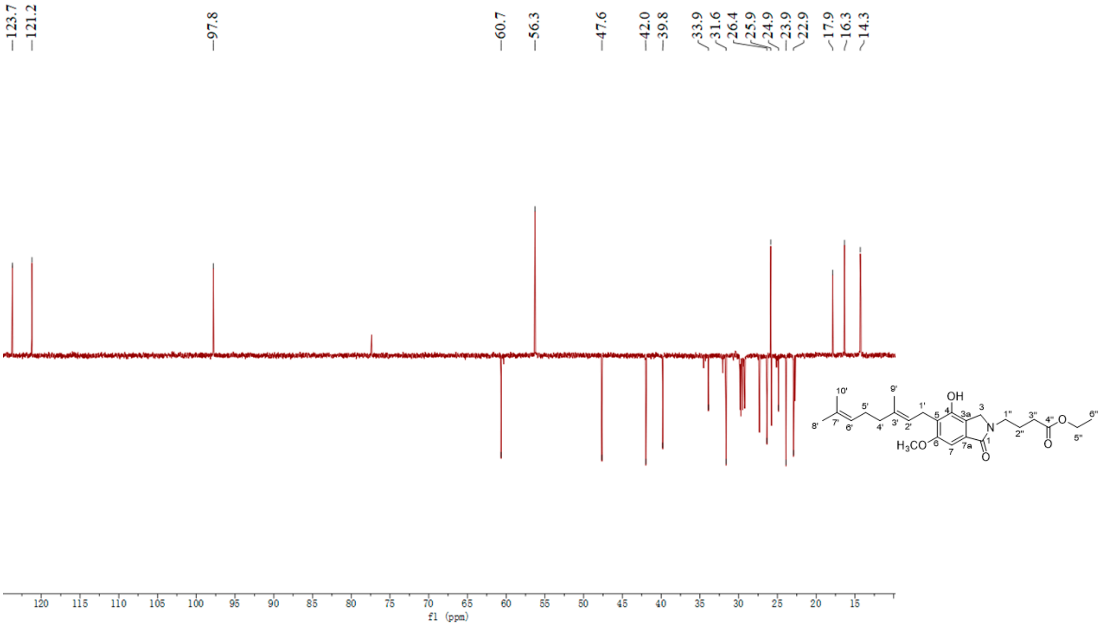


Figure S7.$^{1}$H-$^{1}$H NMR spectrum of compound **1** in $CDCl_3$.

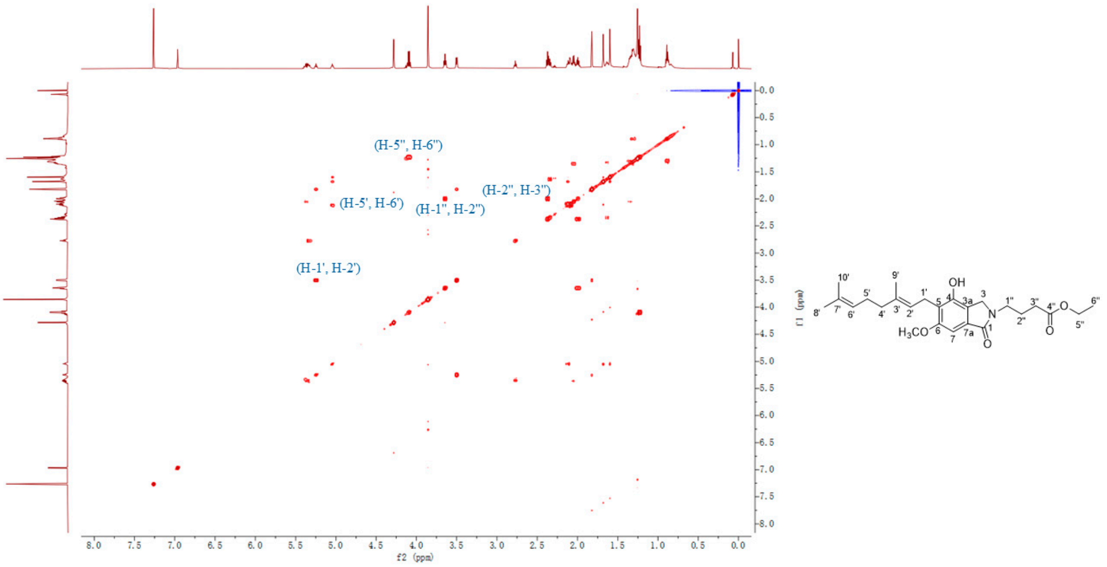

Figure S8. HSQC spectrum of compound **1** in $CDCl_3$.

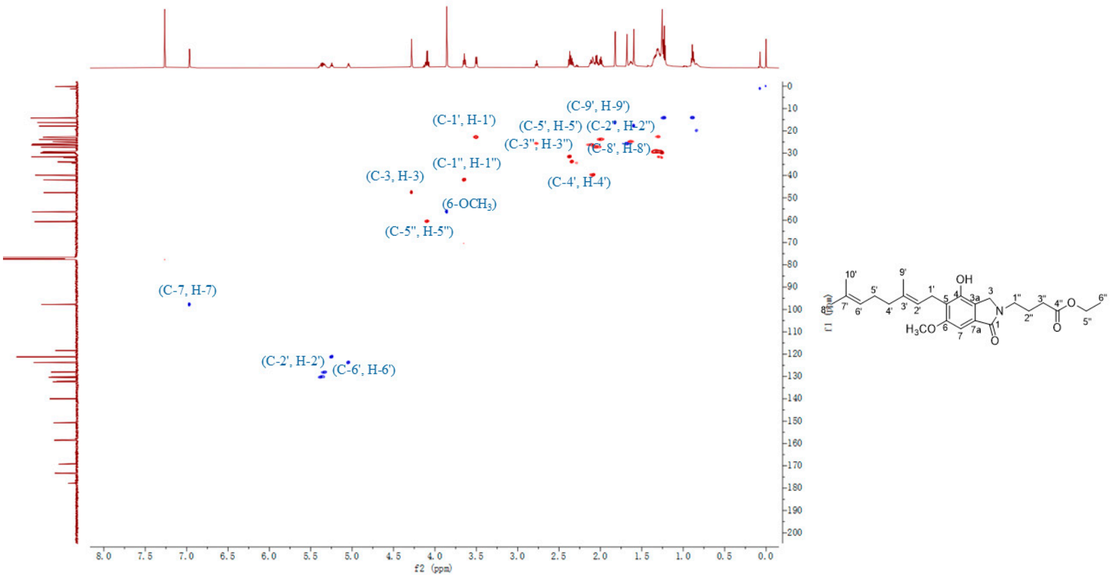


Figure S9. HMBC spectrum of compound **1** in $CDCl_3$.

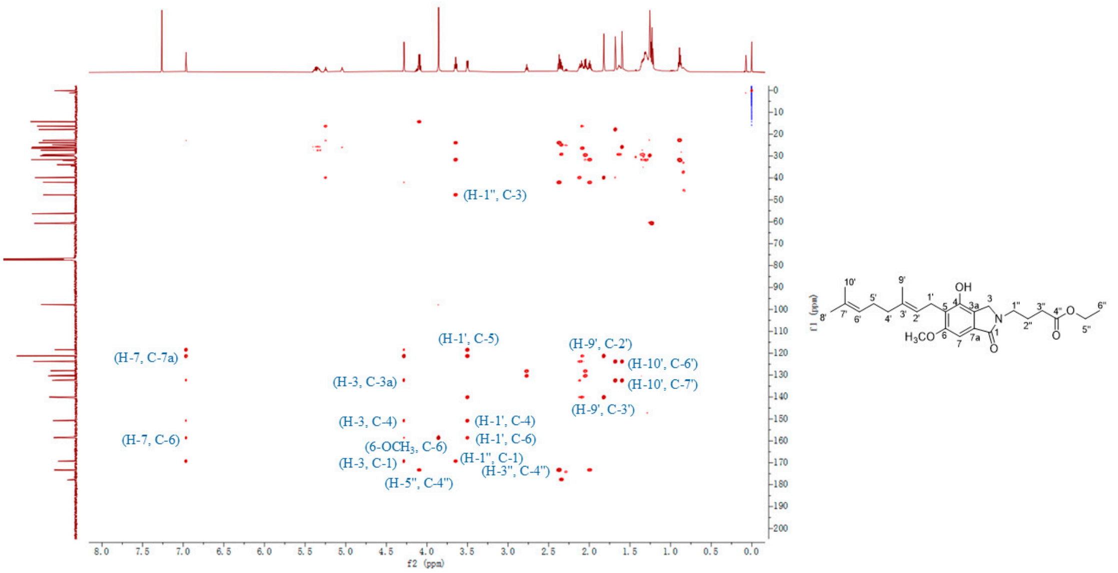

Figure S10. NOESY spectrum of compound **1** in $CDCl_3$.

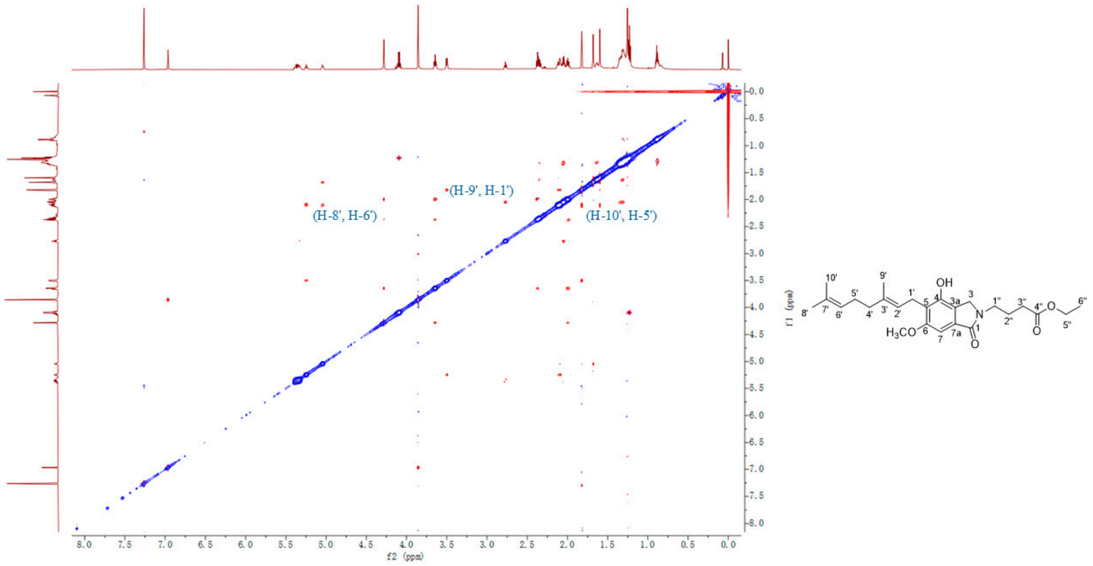


Figure S11. HRESIMS spectrum of compound **2**.

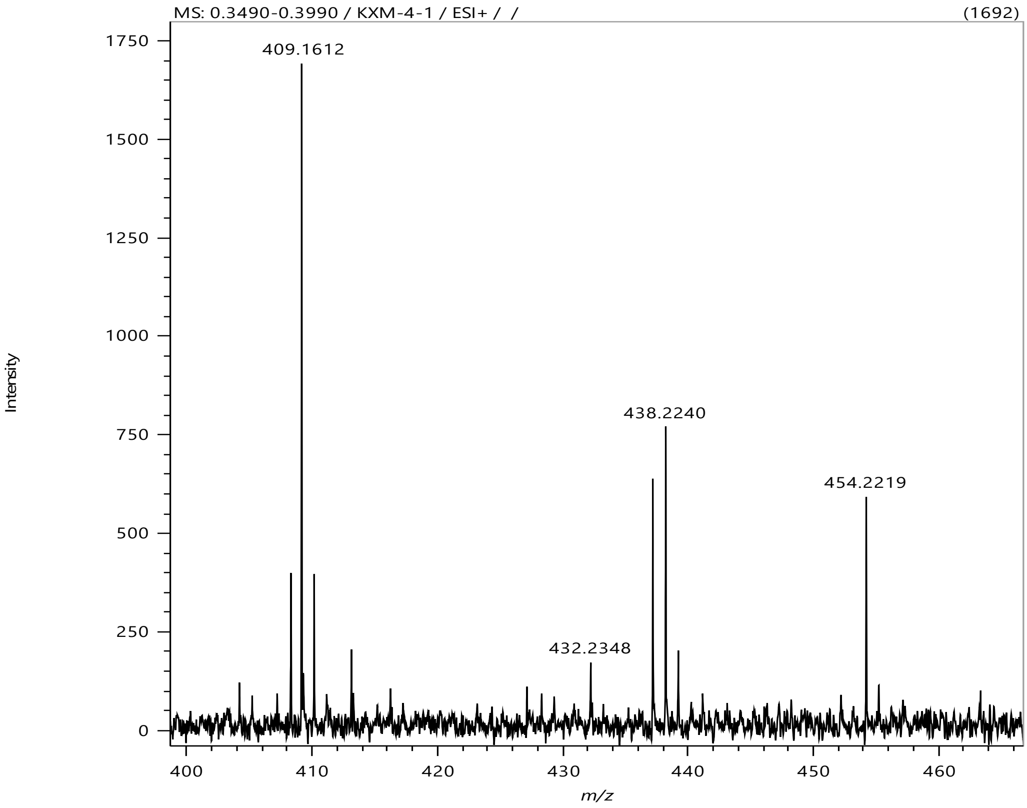


| Mass | Intensity | Formula | Calculated Mass | Mass Difference [ppm] | DBE |
|---|---|---|---|---|---|
| 438.22401 | 770.96 | C24 H33 N O5 Na | 438.22509 | -2.47 | 8.5 |

Figure S12. IR spectrum of compound **2**.

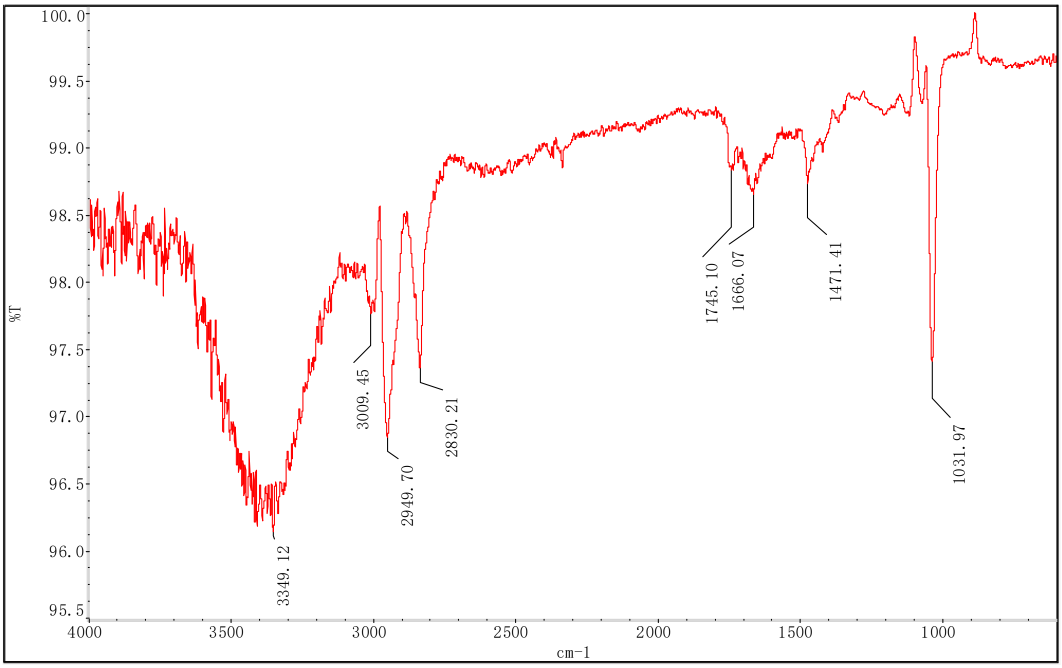

Figure S13. UV spectrum of compound **2**.

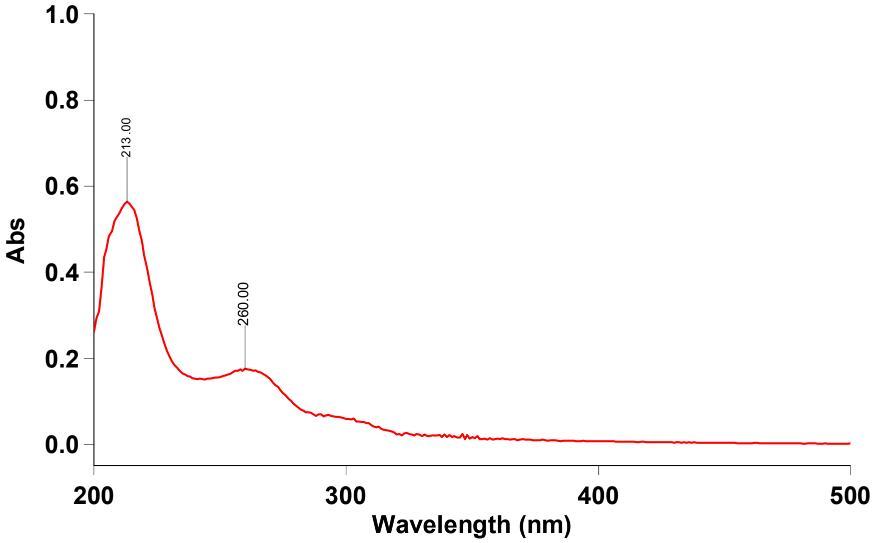


# Scan Analysis Report

Report Time : !″ 05 #$ 02:38:24 PM 2024
Method:
Batch: E:\YZR\20240105\KXM-4.DSW
Software version: 3.00(339)
Operator:

Sample Name: KXM-4 0.008mg-mL

| | |
|---|---|
| Collection Time | 2024-1-5 14:37:17 |

Peak Table

| | |
|---|---|
| Peak Style | Peaks |
| Peak Threshold | 0.0040 |
| Range | 500.00nm to 200.00nm |

| Wavelength (nm) | Abs |
|---|---|
| 260.00 | 0.175 |
| 213.00 | 0.564 |

Figure S14. $^{1}$H NMR spectrum of compound **2** in $CDCl_3$.

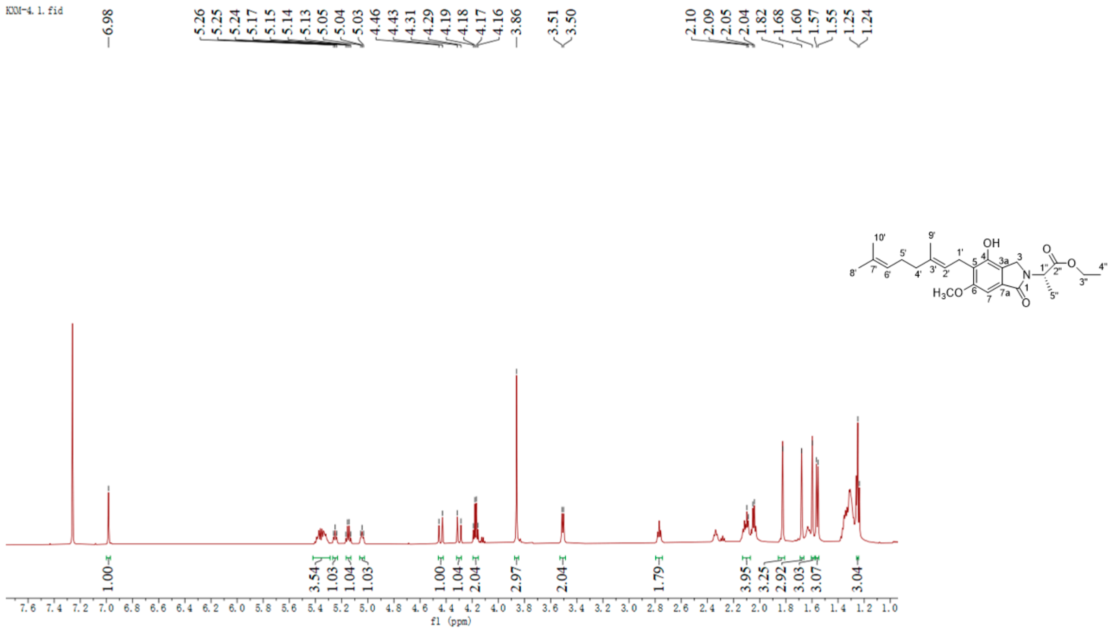


Figure S15. $^{13}$C NMR spectrum of compound **2** in $CDCl_3$.

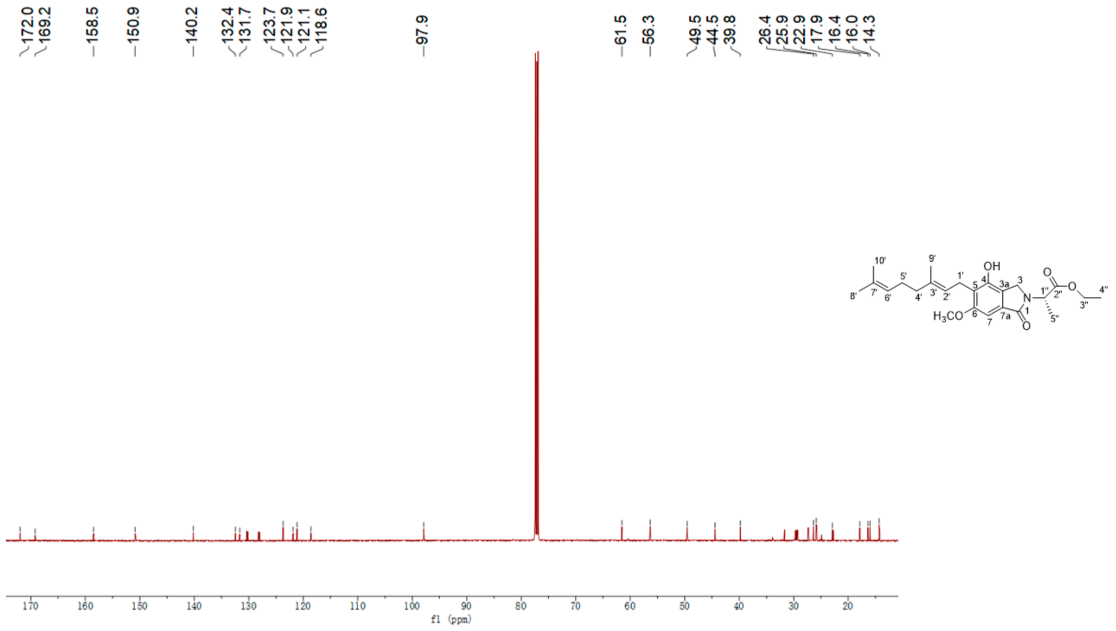

Figure S16. DEPT135 spectrum of compound **2** in $CDCl_3$.

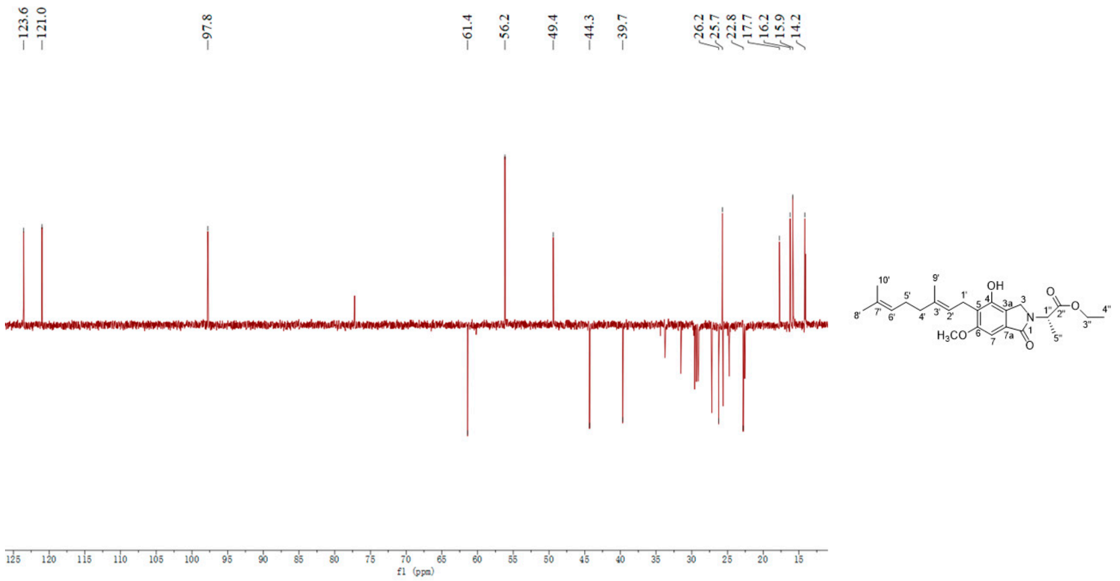


Figure S17. $^1H$-$^1H$ NMR spectrum of compound **2** in $CDCl_3$.

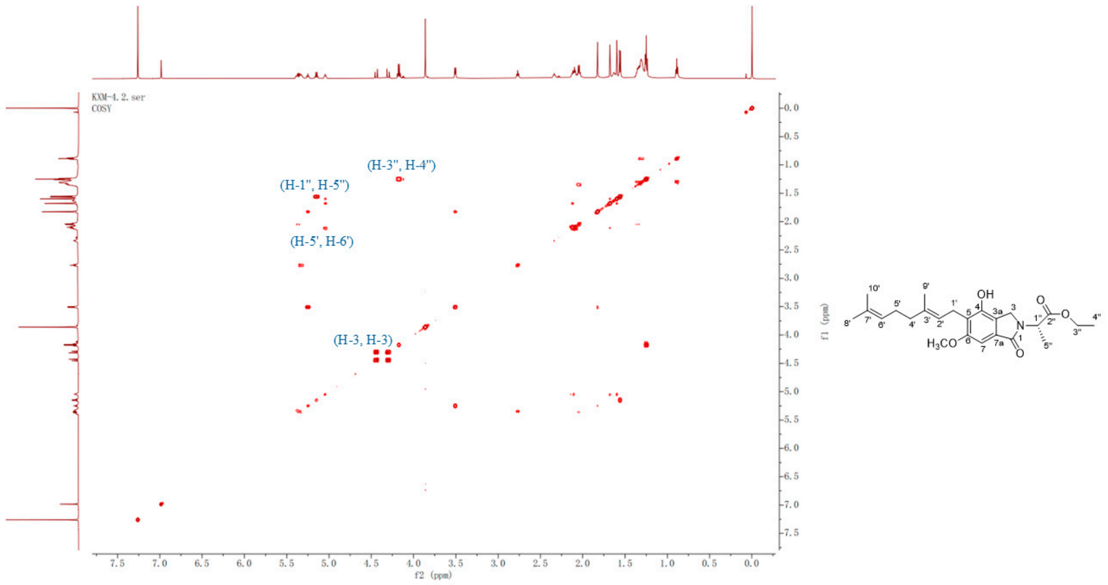

Figure S18. HSQC spectrum of compound **2** in $CDCl_3$.

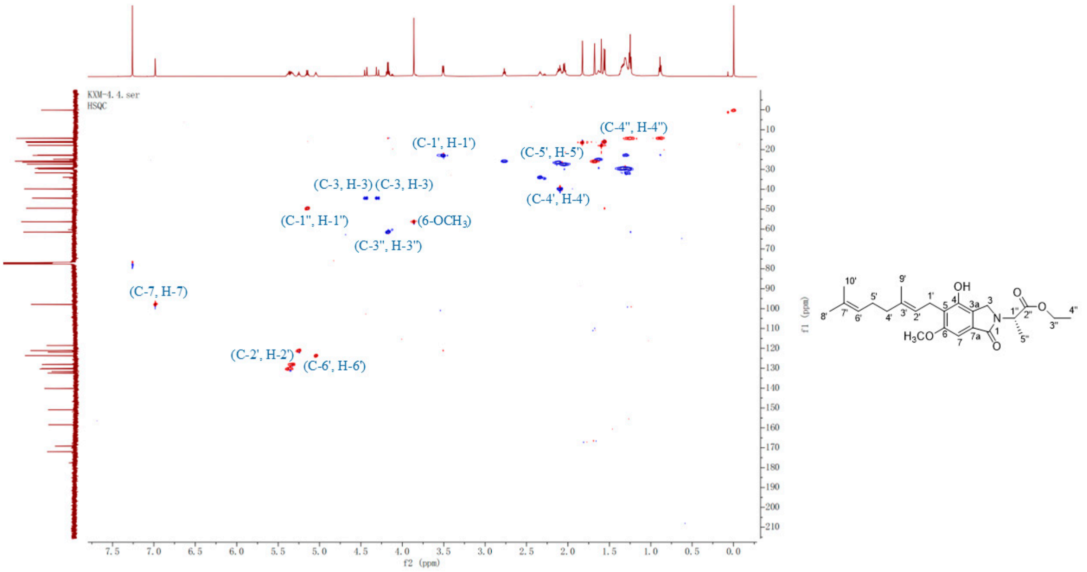


Figure S19. HMBC spectrum of compound **2** in $CDCl_3$.

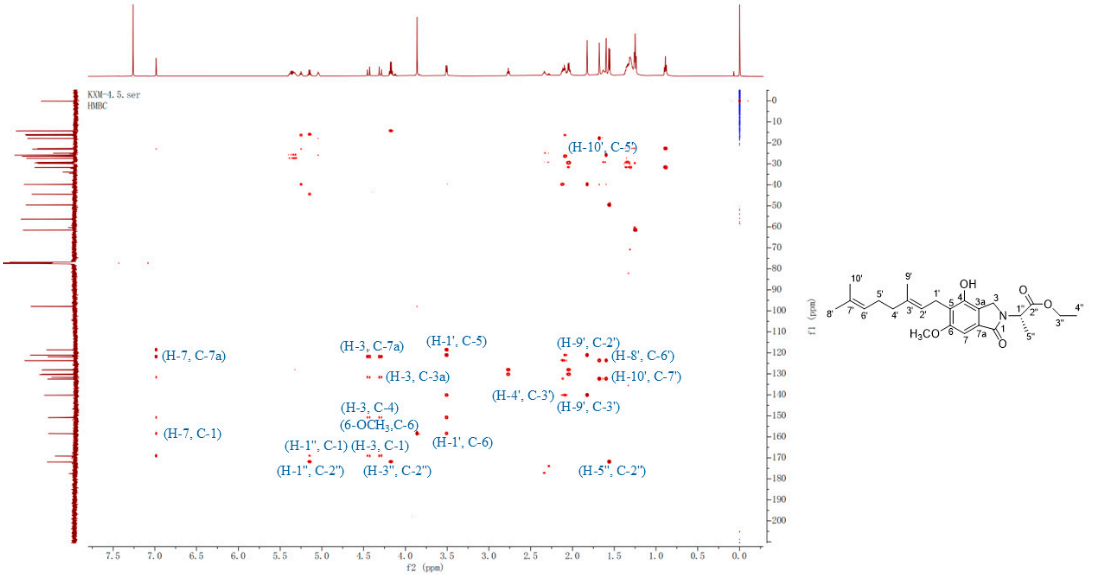

Figure S20. NOESY spectrum of compound **2** in $CDCl_3$.

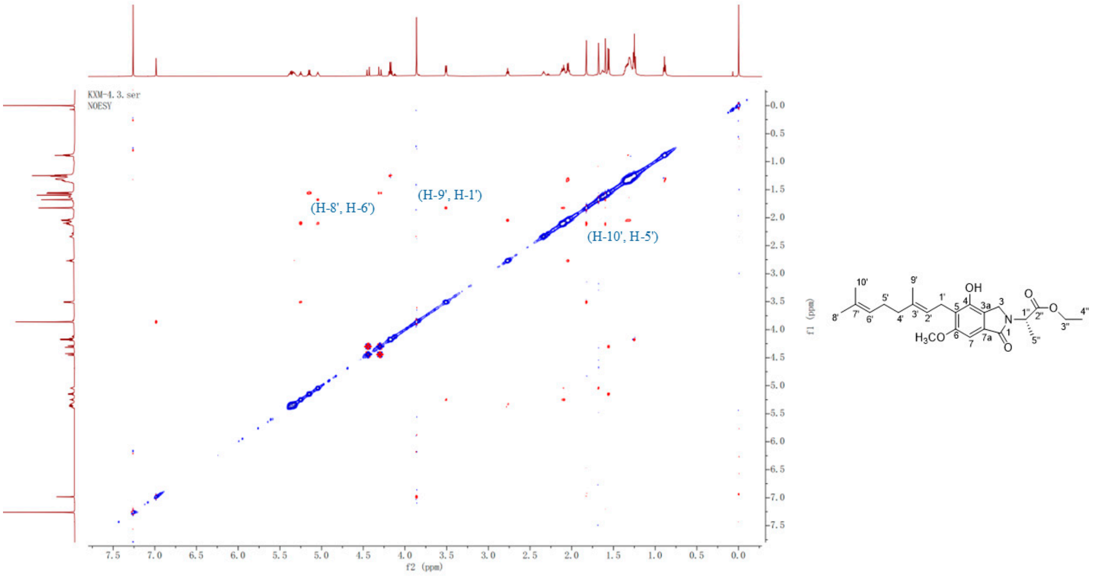


Figure S21. HRESIMS spectrum of compound **6**.

| Mass | Intensity | Formula | Calculated Mass | Mass Difference [ppm] | DBE |
|---|---|---|---|---|---|
| 279.08762 | 244.41 | C15 H14 N O3 Na | 279.08769 | -0.24 | 9.0 |
| | | C14 H15 O6 | 279.08741 | 0.74 | 7.5 |

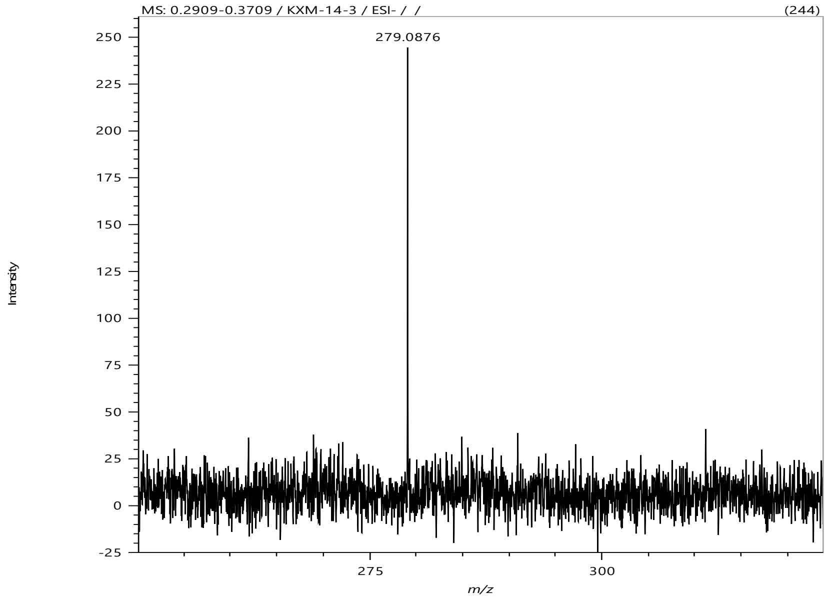

Figure S22. IR spectrum of compound **6**.

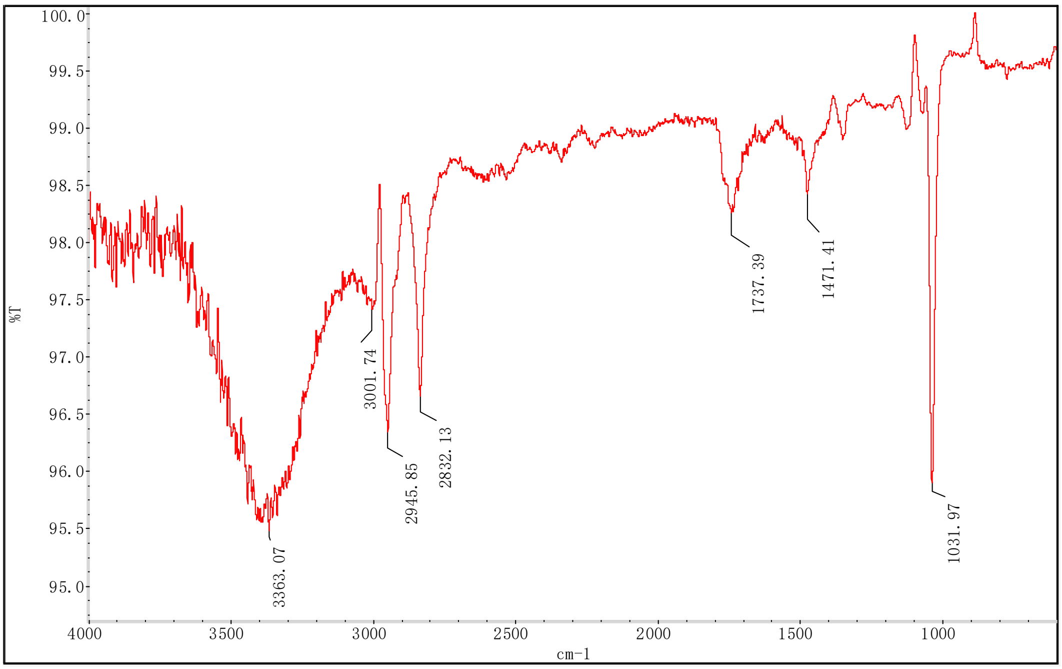

Figure S23. UV spectrum of compound **6**.

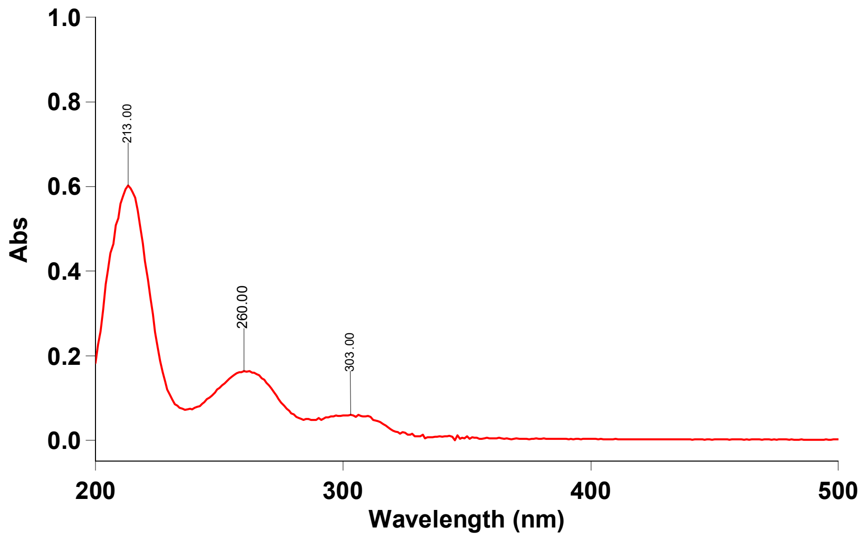


# Scan Analysis Report

Report Time : !" 05 #$ 02:40:20 PM 2024
Method:
Batch: E:\YZR\20240105\KXM-14.DSW
Software version: 3.00(339)
Operator:

Sample Name: KXM-14 0.005mg-mL

| | |
|---|---|
| Collection Time | 2024-1-5 14:42:52 |

Peak Table

| | |
|---|---|
| Peak Style | Peaks |
| Peak Threshold | 0.0040 |
| Range | 500.00nm to 200.00nm |

| Wavelength (nm) | Abs |
|---|---|
| 303.00 | 0.060 |
| 260.00 | 0.163 |
| 213.00 | 0.603 |

Figure S24. $^{1}$H NMR spectrum of compound **6** in $CDCl_3$.

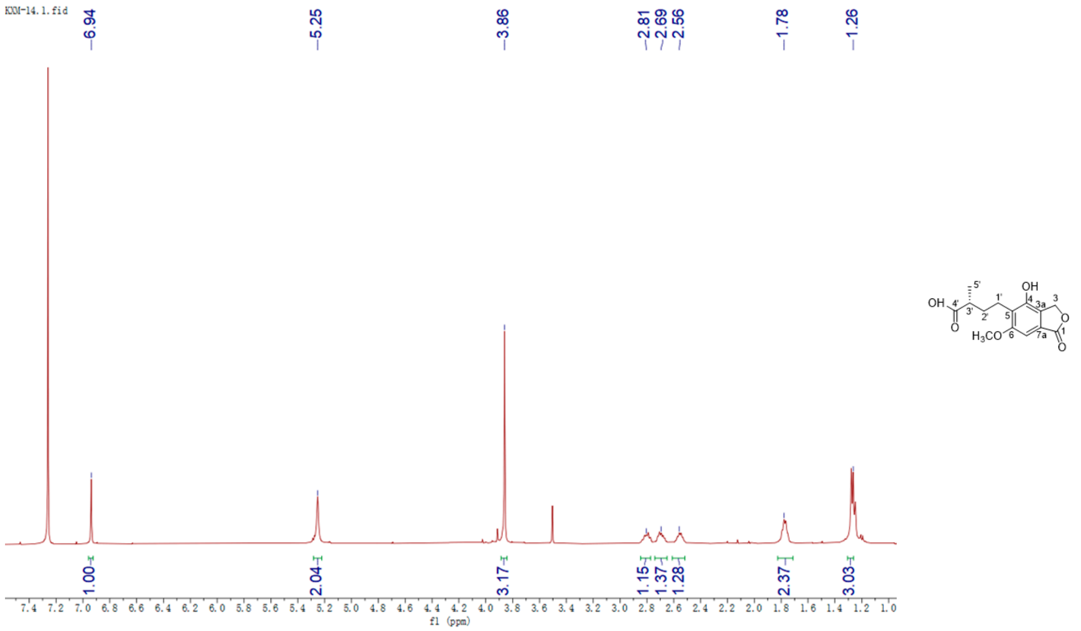


Figure S25. $^{13}$C NMR spectrum of compound **6** in $CDCl_3$.

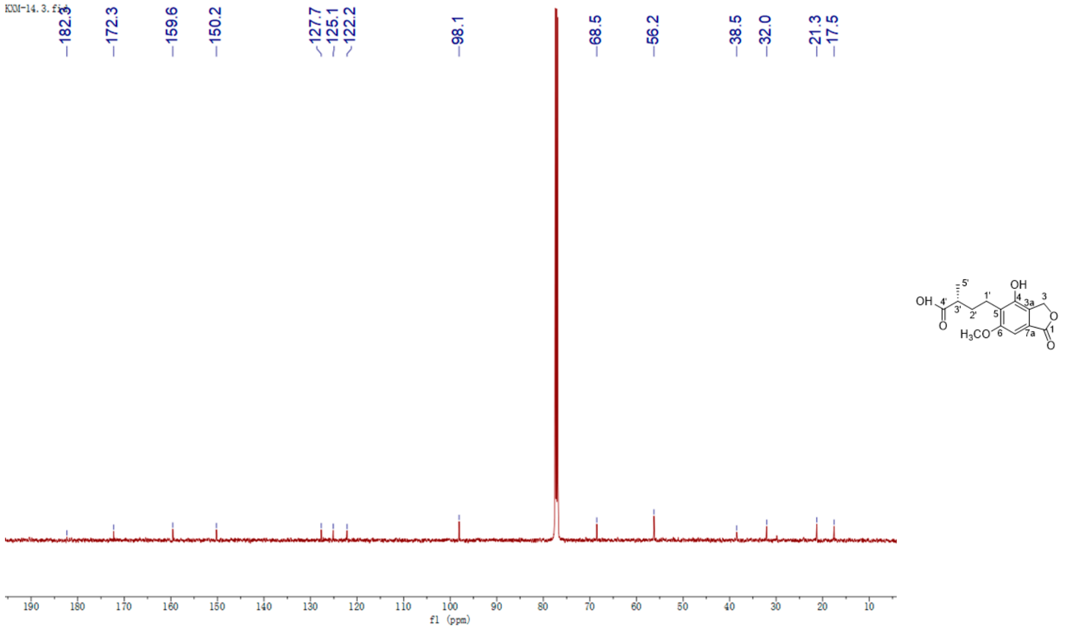

Figure S26. DEPT135 spectrum of compound **6** in $CDCl_3$.

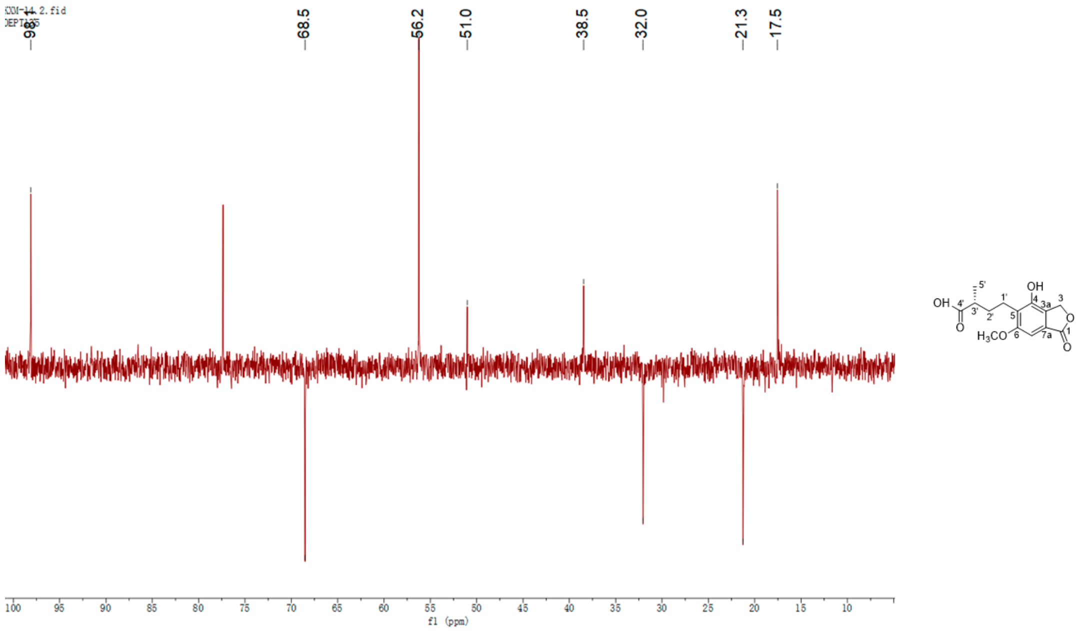


Figure S27. HSQC spectrum of compound **6** in $CDCl_3$.

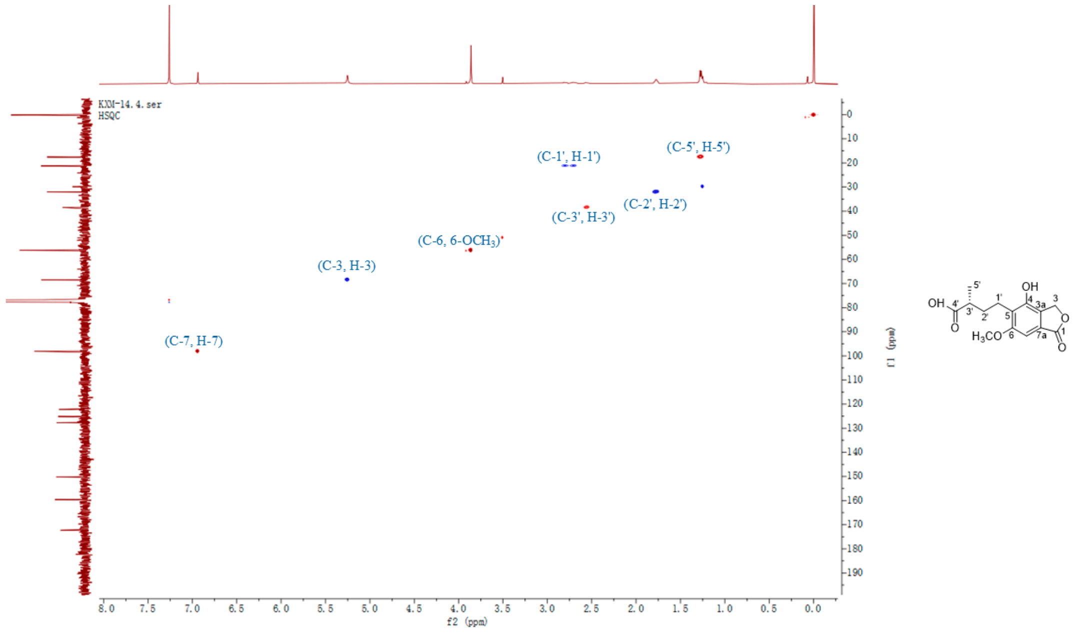

Figure S28. $^1H$-$^1H$ NMR spectrum of compound **6** in $CDCl_3$.

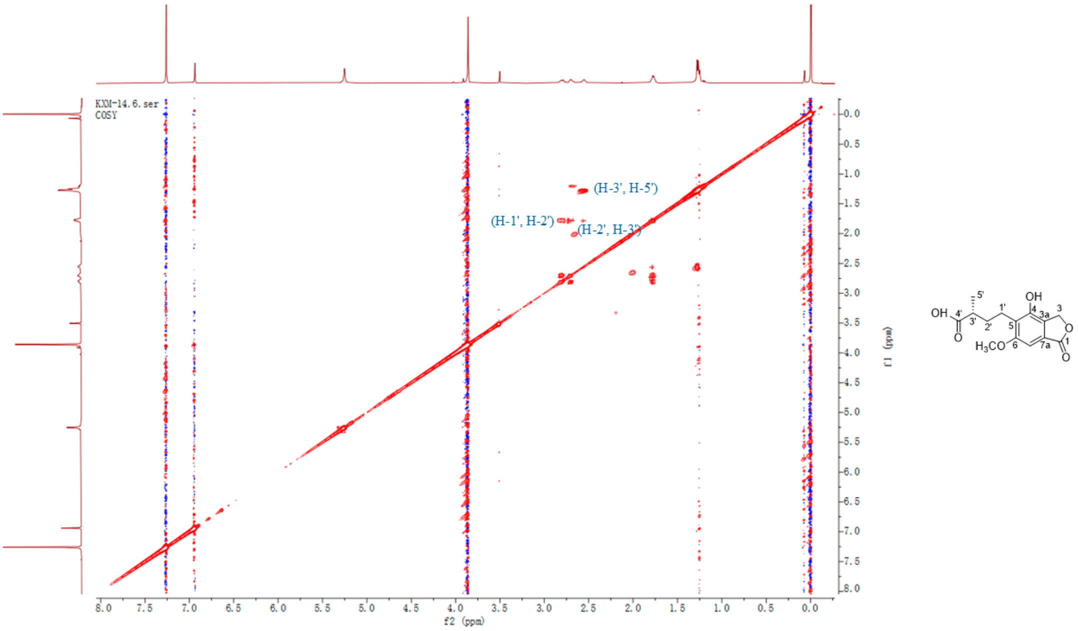


Figure S29. HMBC spectrum of compound **6** in $CDCl_3$.

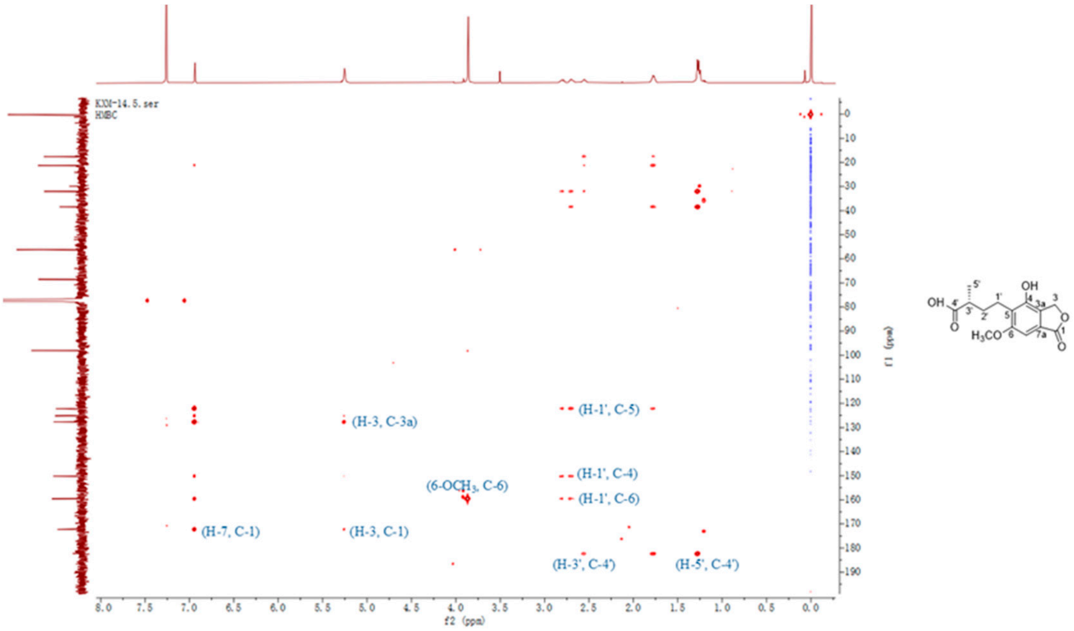

Figure S30. NOESY spectrum of compound **6** in $CDCl_3$.

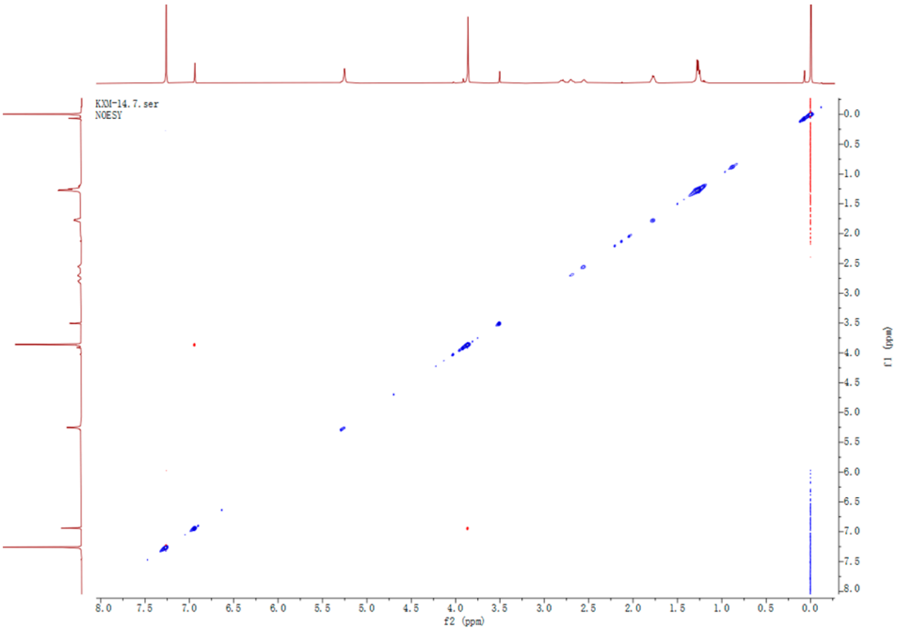


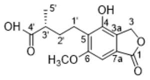

Figure S31. Molecular docking between compounds (**1**–**8** and acarbose) and *α*-glucosidase.

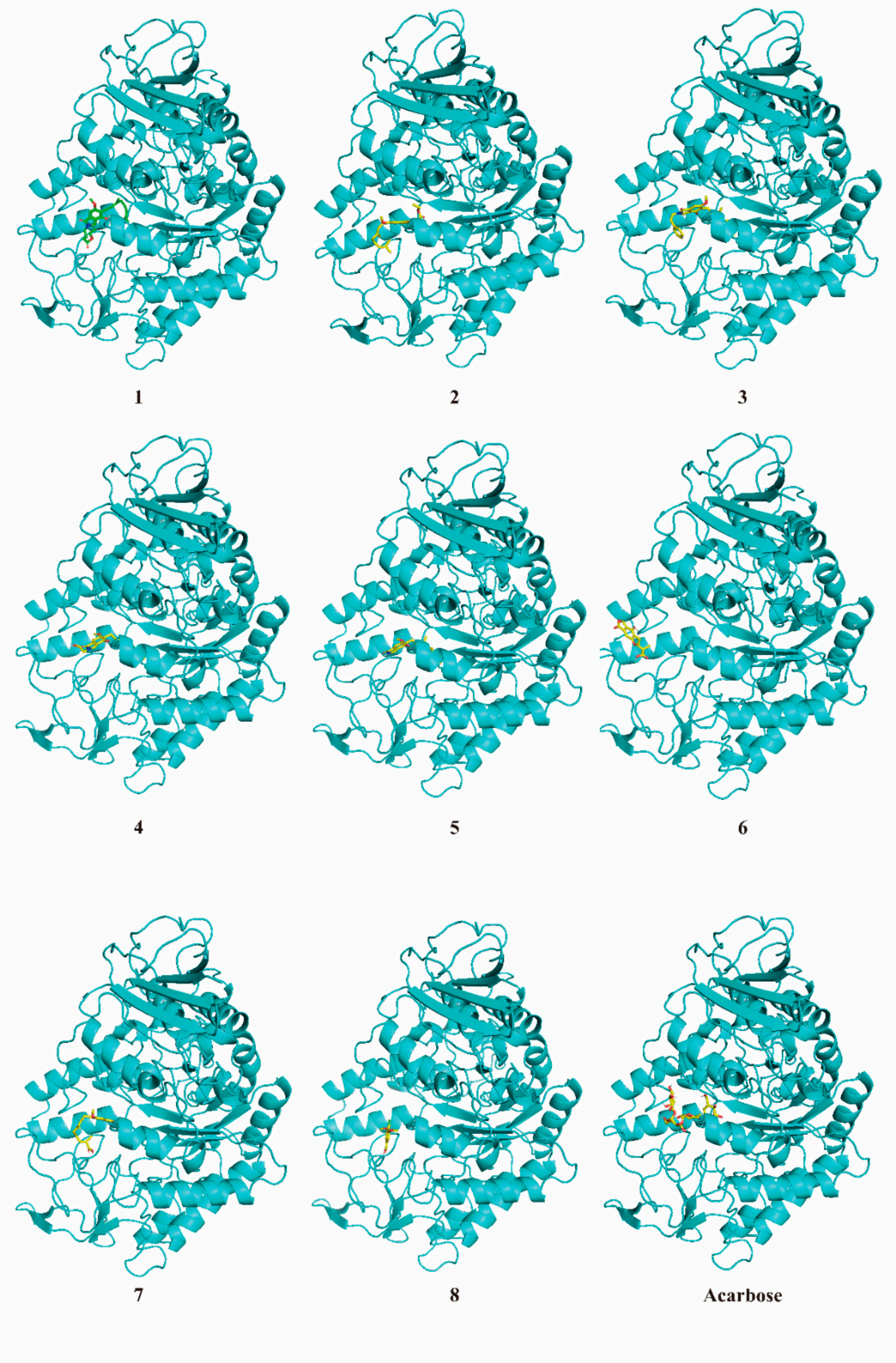

Figure S32. Interactions between acarbose and $\alpha$-glucosidase.

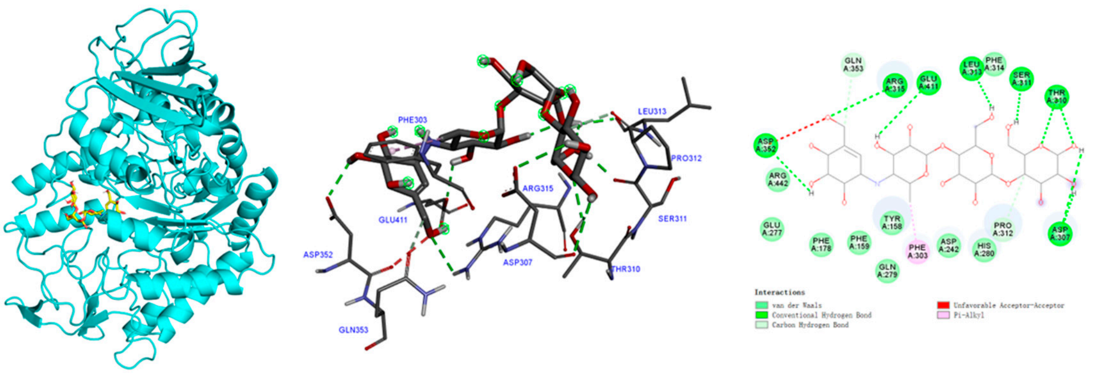


Figure S33. Conformational change of the N-Terminal Residues 4-12 (8261st in purple and 8331st in blue).

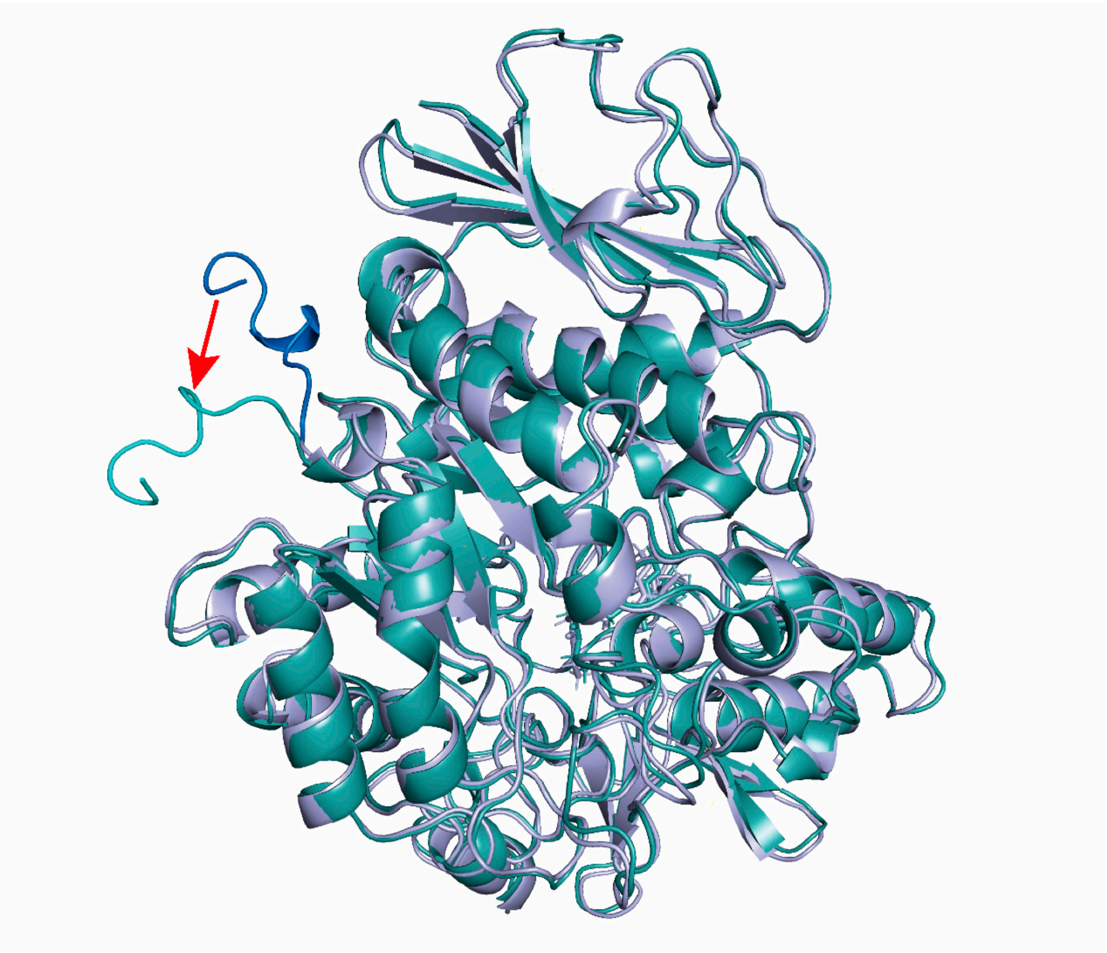

Figure S34. Adjusted RMSD of the **2**-3A4A complex (excluding flexible N-terminal residues 4–12, SSAHPTETP).

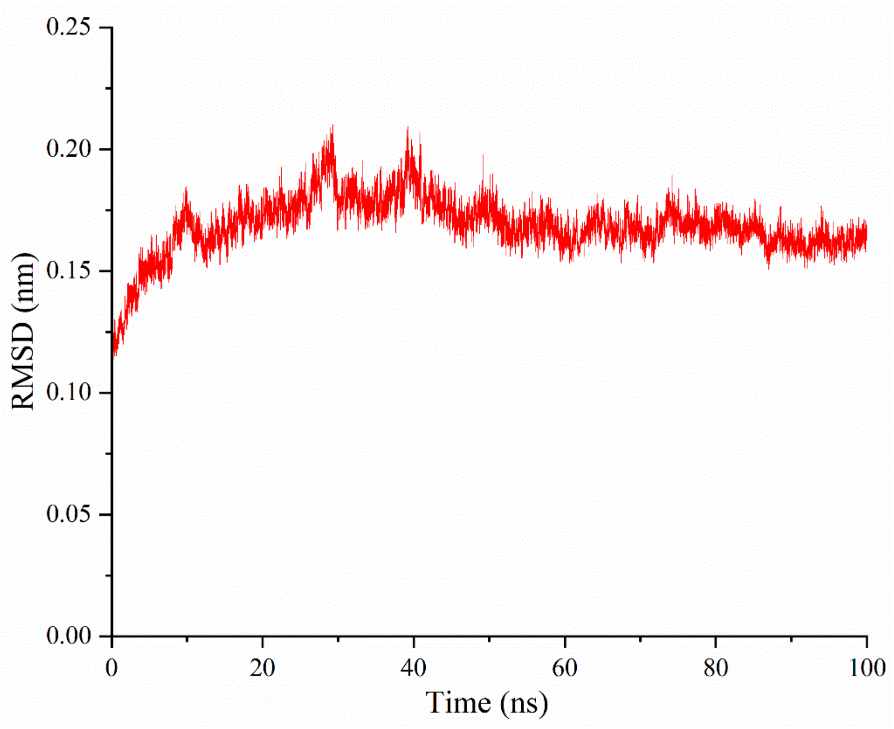

Figure S35 Specific binding affinity by SPR.

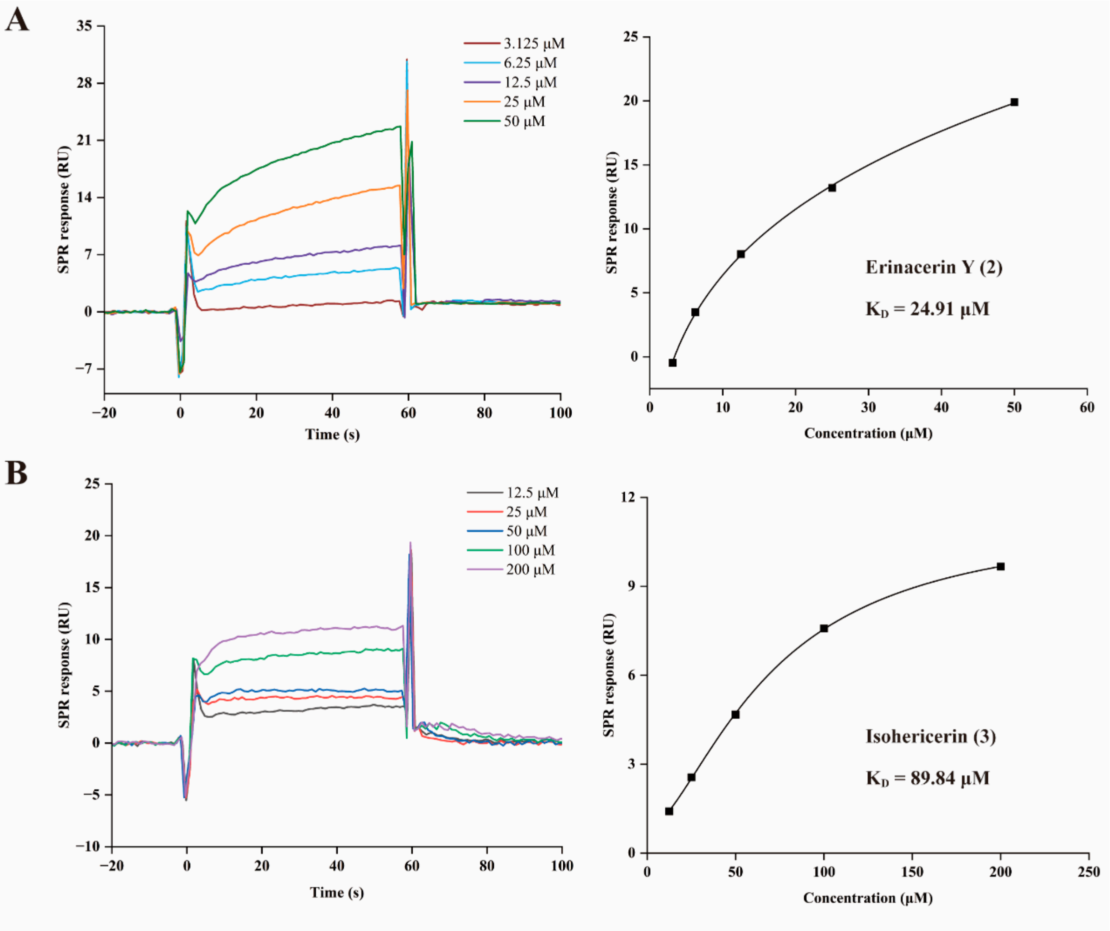


(A) SPR characterization of the affinity (binding sensorgram (left) and normalized steady-state binding curves (right)) between **2** and the $\alpha$-glucosidase, increasing concentrations of **2** from 3.125 $\mu$M to 50 $\mu$M were injected over enzyme surface. (B) SPR characterization of the affinity (binding sensorgram (left) and normalized steady-state binding curves (right)) between **3** and the $\alpha$-glucosidase, increasing concentrations of **3** from 12.5 $\mu$M to 200 $\mu$M were injected over enzyme surface.

Table S1. The molecular docking results for 3A4A with compounds**1**−**8** and acarbose.

| Receptor | Ligands | Docking Score (kcal/mol) |
|---|---|---|
| | **1** | −7.985 |
| $\alpha$-Glucosidase | **2** | −9.991 |
| | **3** | −8.588 |

| | |
|---|---|
| **4** | −7.923 |
| **5** | −8.062 |
| **6** | −7.373 |
| **7** | −7.473 |
| **8** | −5.796 |
| **Acarbose** | −8.442 |
